# Chronic immune activation and gut barrier dysfunction is associated with neuroinflammation in ART-suppressed SIV+ rhesus macaques

**DOI:** 10.1371/journal.ppat.1011290

**Published:** 2023-03-29

**Authors:** Sarah J. Byrnes, Kathleen Busman-Sahay, Thomas A. Angelovich, Skyler Younger, Sol Taylor-Brill, Michael Nekorchuk, Stephen Bondoc, Rachel Dannay, Margaret Terry, Catherine R. Cochrane, Trisha A. Jenkins, Michael Roche, Claire Deleage, Steven E. Bosinger, Mirko Paiardini, Bruce J. Brew, Jacob D. Estes, Melissa J. Churchill

**Affiliations:** 1 School of Health and Biomedical Sciences, RMIT University, Melbourne, Australia; 2 Vaccine & Gene Therapy Institute, Oregon Health & Science University, Portland, Oregon, United States of America; 3 Division of Pathobiology and Immunology, Oregon National Primate Research Center, Oregon Health & Science University, Portland, Oregon, United States of America; 4 Life Science, Burnet Institute, Melbourne, Australia; 5 Department of Infectious Diseases, The Peter Doherty Institute for Infection and Immunity, The University of Melbourne, Melbourne, Australia; 6 AIDS and Cancer Virus Program, Leidos Biomedical Research Inc., Frederick National Laboratory for Cancer Research, Frederick, Maryland, United States of America; 7 Division of Microbiology and Immunology, Emory National Primate Research Center, Emory University, Atlanta, Georgia, United States of America; 8 Department of Pathology and Laboratory Medicine, Emory University School of Medicine, Atlanta, Georgia, United States of America; 9 Peter Duncan Neurosciences Unit, Departments of Neurology and Immunology St Vincent’s Hospital, University of New South Wales and University of Notre Dame, Sydney, New South Wales, Australia; 10 Departments of Microbiology and Medicine, Monash University, Clayton, Australia; Vaccine Research Center, UNITED STATES

## Abstract

HIV-associated neurocognitive disorders (HAND) affect ~40% of virally suppressed people with HIV (PWH), however, the precise viral dependent and independent changes to the brain are unclear. Here we characterized the CNS reservoir and immune environment of SIV-infected (SIV+) rhesus macaques during acute (n = 4), chronic (n = 12) or ART-suppressed SIV infection (n = 11). Multiplex immunofluorescence for markers of SIV infection (vRNA/vDNA) and immune activation was performed on frontal cortex and matched colon tissue. SIV+ animals contained detectable viral DNA+ cells that were not reduced in the frontal cortex or the gut by ART, supporting the presence of a stable viral reservoir in these compartments. SIV+ animals had impaired blood brain barrier (BBB) integrity and heightened levels of astrocytes or myeloid cells expressing antiviral, anti-inflammatory or oxidative stress markers which were not abrogated by ART. Neuroinflammation and BBB dysfunction correlated with measures of viremia and immune activation in the gut. Furthermore, SIV-uninfected animals with experimentally induced gut damage and colitis showed a similar immune activation profile in the frontal cortex to those of SIV-infected animals, supporting the role of chronic gut damage as an independent source of neuroinflammation. Together, these findings implicate gut-associated immune activation/damage as a significant contributor to neuroinflammation in ART-suppressed HIV/SIV infection which may drive HAND pathogenesis.

## Author summary

Currently no cure for HIV exists and people with HIV experience long term cognitive issues which significantly impacts their quality of life. The mechanisms driving cognitive impairment are unclear and difficult to study in humans, but likely relate to (i) HIV hiding within the brain causing localised brain damage and (ii) a chronic inflammatory state in the body that may spill over into the brain. Here we used the well-characterized Simian Immunodeficiency Virus non-human primate model of HIV to define the presence and effects of SIV in the brain. We demonstrate that the frontal cortex of the brain of SIV-infected animals remains in an activated and inflammatory state, despite long term treatment with anti-HIV therapies. Intriguingly, we show that whilst SIV remains in the frontal cortex despite long-term anti-HIV therapies, the virus does not appear to be the sole driver of inflammation in this tissue. Instead, chronic inflammation related to damage of the gastrointestinal tract was associated with changes in the immune environment in the frontal cortex. We further demonstrated that gut damage alone can induce immune activation in the frontal cortex in a similar manner to that seen in animals with SIV. Together, these findings indicate that damage to the gut can contribute to activation of brain cells, which may contribute to brain damage during SIV, and by extension, HIV infection.

## Introduction

Currently ~40% of people with HIV (PWH) develop a form of HIV-associated neurocognitive disorder (HAND) despite effective viral suppression with antiretroviral therapies (ART) [1,2]. These disorders can impair cognitive and motor functions including causing problems with social interactions, independence and daily activities (i.e. internet use and driving [3]) and have been associated with reduced life expectancy [4]. The mechanisms driving HAND pathogenesis remain ill-defined, but persistent viral infection in the brain and neuroinflammation driven by ongoing systemic immune activation penetrating the brain are thought to play crucial roles.

HIV infects the central nervous system (CNS) during acute stages of infection and disseminates to perivascular macrophages, microglia, pericytes and astrocytes which is associated with neuronal cell death, loss of brain volume and tissue damage [5–7]. High HIV plasma/CSF viral load and breakdown of the blood brain barrier (BBB) are associated with the pathogenesis of severe forms of HAND and/or HIV-encephalitis (HIVE) [8–11]. However, the role of both ongoing viremia and immune activation in the pathogenesis of milder cognitive impairment in ART-suppressed PWH is unclear. We recently demonstrated that ART-suppressed PWH harbor intact potentially replication competent viral genomes in the brain [12], which may induce viral mediated damage in the CNS. Clinical evidence of reduced brain volumes, heightened measures of neuroinflammation/neurodegradation (as measured by fMRI analysis and CSF/plasma biomarkers), reduced synaptic density, and in some cases progressive cognitive disease severity in ART-suppressed PWH [13–15] further demonstrate ongoing pathology in the brain of ART-suppressed PWH. However, how the cellular environment within the brain of ART-suppressed PWH is altered and what role viral reservoirs in the brain play in disease pathogenesis is unclear and difficult to study in humans.

Simian immunodeficiency virus (SIV) infection models in rhesus macaques (RMs) are fundamental tools in neuroHIV research allowing for a comprehensive analysis of the mechanistic pathways affecting the brain with greater flexibility and depth than human *ex vivo* studies alone [16]. SIV-infected (SIV+) RMs mimic key pathological features of HIV pathogenesis in humans, including CD4+ T-cell depletion, chronic systemic inflammation, lymphoid and gut tissue pathology and neuropathology [17,18]. To date, SIV infection studies using neuro-adapted SIV strains have provided significant insight into SIV/HIV neuropathogenesis and encephalitis, demonstrating that SIV rapidly infects the brain via infected monocytes and T cells and spreads to perivascular macrophages, microglia, astrocytes and pericytes [19–22]. Moreover, CNS infection and encephalitis is associated with a spike in localized immune activation in the brain, suggestive of an active immune response to virus. Studies using non-accelerated SIV strains (e.g. SIVmac239 linages), which may more closely model natural CNS disease pathogenesis in PWH, further support ongoing immune activation in the CNS during infection [23–25] and a stable reservoir of SIV that is not abrogated by ART [26]. However, comparatively little is known about the immune environment of the brain in chronically SIV-infected ART-treated animals, or the role that the viral reservoir plays in driving immune activation, particularly at a cellular level.

Another potential confounding driver of HIV/SIV neuropathogenesis is systemic inflammation/immune activation as measures of inflammation in the plasma/CSF of PWH or animal models have been associated with immune activation in the brain [27–29]. Systemic inflammation is a hallmark of HIV/SIV and is thought to be caused, in part, by persistent viral reservoirs in the gut that are not eradicated by ART and early damage to the gastrointestinal (GI) epithelial barrier leading to “leaky gut” whereby microbes/microbial products (e.g. lipopolysaccharide) translocate into the bloodstream driving immune activation [26,30–32]. Gut damage alone has independently been linked to neuroinflammation in mouse models [33]. However, the role and extent by which systemic inflammation penetrates the brain and contributes to neuroinflammation in HIV/SIV is unclear. Moreover, whether gut damage alone can drive neuroinflammation independent of SIV is unknown and difficult to assess in humans.

Here we utilized spatial *in situ* multiplex immunofluorescence analyses to comprehensively assess the viral reservoir, integrity of the BBB, and ongoing immune activation/inflammation at a cellular level in the frontal cortex and gut of SIV+ animals during acute, chronic, and ART-suppressed SIV infection. Furthermore, we utilized a novel model of gut damage in SIV-uninfected RMs which mimics that seen in SIV infection [34] to determine whether GI damage without concomitant SIV infection independently contributes to neuroinflammation.

## Results

### Nonhuman primate cohort

Frontal cortex and matched large bowel gut tissues (where available) from SIV infected and uninfected RMs was collected from previously conducted studies performed under institutional approval (Table 1) [26,34–37]. RMs were infected with SIVmac239 or SIVmac251 and either virally suppressed with ART (n = 11) or remained viremic (i.e. not virally suppressed; n = 16; Table 1). SIVmac239 and SIVmac251 were selected as these strains induce a mild neuropathology early after infection, which more closely mimics natural HIV infection compared to other neuroinvasive strains [38]. Untreated animals were sacrificed during acute [SIV+ Acute; n = 4; sacrificed 4 weeks post infection (WPI); median plasma viral load: 1,395,000 SIV RNA copies/mL] or chronic infection [SIV+ Chronic; n = 12; sacrificed a median of 53.5 WPI; median plasma viral load: 200,904 SIV RNA copies/mL; median CD4+ T cells: 382.5 cells/mm^3^]. ART- suppressed animals [SIV+ VS; n = 11; median treatment time: 33 weeks; median plasma viral load: ≤70 SIV RNA copies/mL; median CD4+ T cells: 552 cells/mm^3^] were sacrificed after a median 41 WPI and 33 weeks of ART treatment [26,34–37]. Four uninfected animals were included as controls. To assess the neuroinflammatory implications of chronic colitis an additional four uninfected animals were treated with dextran sulfate sodium (DSS) in their drinking water. Six cycles of DSS treatment (1 cycle = 14 days DSS followed by 14 days normal water) were performed prior to sacrifice and harvesting samples as previously described [34].

**Table 1:** Nonhuman primate cohort.

								ART		Reference
Group	ID	Sex	Site	Virus	CD4 (cells/mm^3^)	Viral load (copies/mL)	WPI	Duration (weeks)	Regimen	
SIV-	30569	F	ONPRC	NEG	ND	-	-	-	-	-
	32135	M	ONPRC	NEG	ND	-	-	-	-	-
	32578	M	ONPRC	NEG	ND	-	-	-	-	-
	33632	F	ONPRC	NEG	ND	-	-	-	-	-
SIV- DSS+	ZL11	F	NIH/NCI	NEG	1,826	-	-	-	-	-
	ZL23	F	NIH/NCI	NEG	593	-	-	-	-	-
	ZL61	M	NIH/NCI	NEG	1,084	-	-	-	-	-
	ZL65	M	NIH/NCI	NEG	948	-	-	-	-	-
Acute SIV+	RMe14	M	YNPRC	SIV_mac239_	ND	1,230,000	4	-	-	-
	ROl14	M	YNPRC	SIV_mac239_	ND	3,460,000	4	-	-	-
	RSb14	M	YNPRC	SIV_mac239_	ND	912,000	4	-	-	-
	RCt14	M	YNPRC	SIV_mac239_	ND	1,560,000	4	-	-	-
Chronic SIV+	RYn10 *	F	YNPRC	SIV_mac239_	132	4,155,157	51	-	-	[1]
	RAy10 *	M	YNPRC	SIV_mac239_	613	1,581,349	57	-	-	[1]
	RGd11 *	M	YNPRC	SIV_mac239_	948	313,422	65	-	-	[1]
	RPt10 *	M	YNPRC	SIV_mac239_	391	14,320	52	-	-	[1]
	RPz10 *	M	YNPRC	SIV_mac239_	99	88,385	55	-	-	[1]
	KKE	F	YNPRC	SIV_mac251_	480	6,160,000	68	-	-	[2]
	RMd5	F	YNPRC	SIV_mac251_	373	424,000	44	-	-	[3]
	RZu4	F	YNPRC	SIV_mac251_	265	737,000	67	-	-	[3]
	R673 ǂ	F	Bioqual	SIV_mac251_	ND	549	20	12	PMPA/FTC/RAL/DRV.r	-
	R674 ǂ	F	Bioqual	SIV_mac251_	ND	874	21	12	PMPA/FTC/RAL/DRV.r	-
	R675 ǂ	F	Bioqual	SIV_mac251_	ND	901	22	12	PMPA/FTC/RAL/DRV.r	-
	RAi13 ǂ	F	Bioqual	SIV_mac251_	374	166	68	60	PMPA/FTC/RAL/MVC, DRV.r	[4]
Virally Suppressed SIV+	HI08	M	NIH/NCI	SIV_mac239_	1,216	70	36	28	PMPA/FTC/DTG/DRV.r	-
	HI34	M	NIH/NCI	SIV_mac239_	1,003	<15	36	28	PMPA/FTC/DTG/DRV.r	-
	ZI17	M	NIH/NCI	SIV_mac239_	910	<15	36	28	PMPA/FTC/DTG/DRV.r	-
	ZI20	M	NIH/NCI	SIV_mac239_	920	<15	36	28	PMPA/FTC/DTG/DRV.r	-
	ZI44	F	NIH/NCI	SIV_mac239_	1,388	70	36	28	PMPA/FTC/DTG/DRV.r	-
	RPa10	F	YNPRC	SIV_mac251_	463	<60	41	33	PMPA/FTC/RAL/DRV.r	[4]
	RWo10	F	YNPRC	SIV_mac251_	614	<60	41	33	PMPA/FTC/RAL/DRV.r	[4]
	RJf13	F	YNPRC	SIV_mac251_	490	<60	55	47	PMPA/FTC/RAL/MVC, DRV.r	[4]
	RPa13	F	YNPRC	SIV_mac251_	728	<60	55	47	PMPA/FTC/RAL/MVC, DRV.r	[4]
	RZm11	F	YNPRC	SIV_mac251_	194	<60	65	57	PMPA/FTC/RAL/MVC, DRV.r	[4]
	RYb13	F	YNPRC	SIV_mac251_	635	<60	68	60	PMPA/FTC/RAL/MVC, DRV.r	[4]

DRV.r: darunavir/ritonavir; DSS: dextran sulfate sodium; FTC: emtricitabine; MVC: maraviroc; ND: Measure not done; PMPA: 9-[2-(Phosphonomethoxy)propyl]adenine; RAL: raltegravir; SIV: simian immunodeficiency virus

* Gut tissue unavailable

ǂ ART-treated, not virally suppressed

### SIV DNA+ and RNA+ cells persist in frontal cortex despite ART

**Fig 1:**
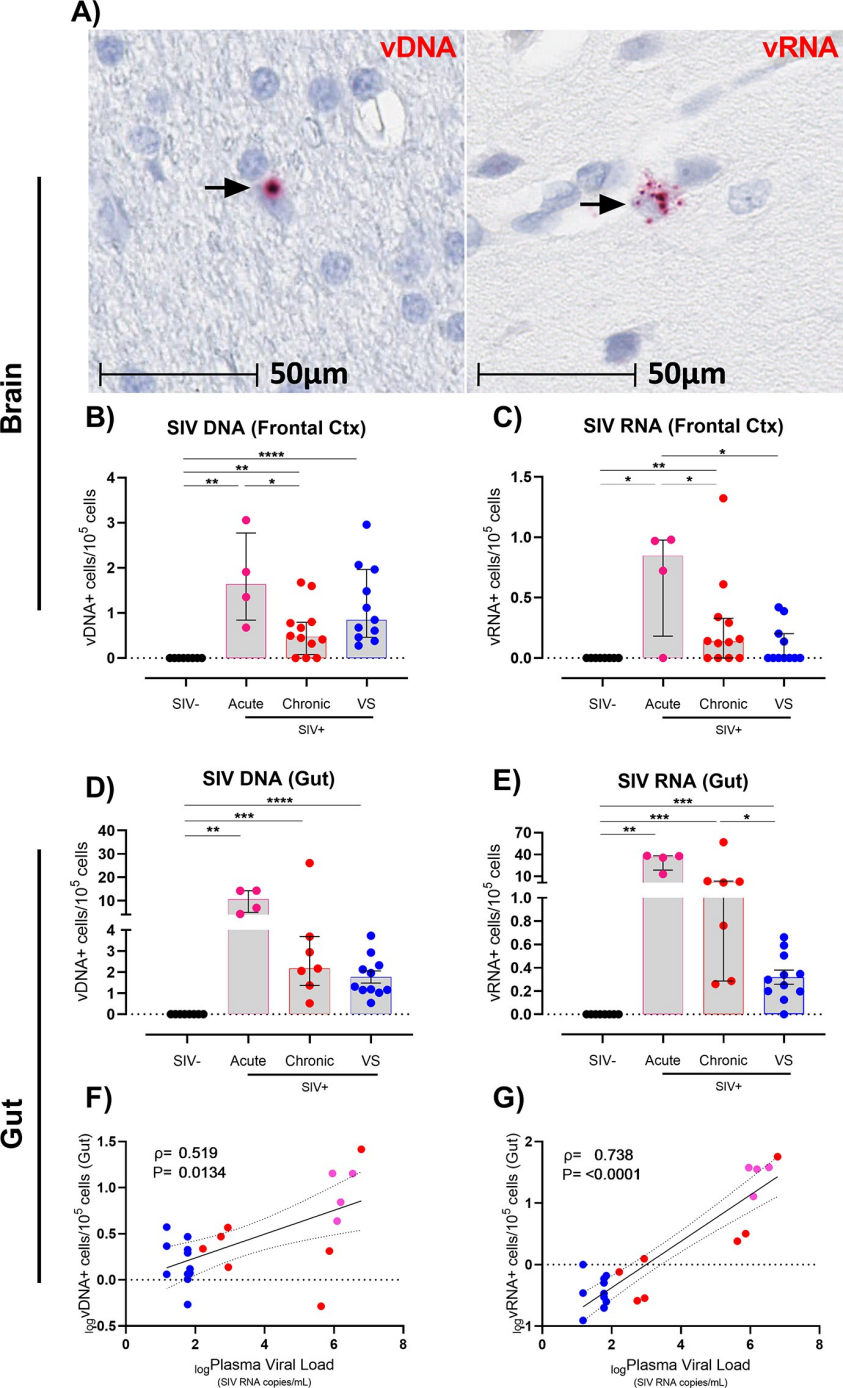
Frequency of vDNA and vRNA cells in frontal cortex and gut tissue from viremic and virally suppressed SIV+ rhesus macaques. **(A)** Representative images of brain cells harbouring vDNA or vRNA (black arrows) detected via DNAscope and RNAscope analysis in frontal cortex brain tissue from chronic SIV+ RMs. Images shown at x60 magnification. Frequency of vDNA+ cells or vRNA+ cells in the **(B and C)** brain or **(D and E)** gut tissue. Comparisons made using Mann-Whitney U test, (*P<0.05, **P<0.01, ***P<0.001). Median and interquartile ranges shown. **(F and G)** Correlative analysis of plasma viral load vs **(F)** vDNA+ or **(G)** vRNA+ cells in the gut (Spearman correlation, P value and Spearman ρ shown). SIV+ virally suppressed RMs (blue circles), SIV+ viremic RMs (red circles), SIV+ acute RMs (pink circles).

To quantify SIV viral DNA (vDNA+) and RNA (vRNA+) cells in the brain of SIV+ RMs, DNAscope and RNAscope were performed on frontal cortex tissue sections (representative images shown in Fig 1A). SIV DNA+ cells were detected in the frontal cortex during acute infection (median: 1.63 vDNA+ cells/10^5^ cells; P<0.01 relative to uninfected controls; Fig 1B) and in chronically infected, untreated animals (median: 0.47 vDNA+ cell/10^5^ cells). Importantly, SIV vDNA+ cells were present in the frontal cortex of virally suppressed animals (median 0.85 vDNA+ cell/10^5^) at similar levels to chronically infected animals, indicating that chronic ART-treatment does not influence CNS reservoir size in agreement with our previous findings [26]. Viral RNA+ cells were present in the frontal cortex of untreated animals during acute and chronic stages of infection (Fig 1C). vRNA+ cells were also detected in some, but not all virally suppressed animals (4/11 animals; Fig 1C).

To determine how the CNS reservoir in SIV-infected animals compared to non-CNS lymphoid reservoirs, SIV vRNA and vDNA was also quantified in matched transverse colon tissue where available (all animals except n = 5 chronic untreated animals, Table 1). SIV vDNA was present in transverse colon tissue in both viremic (acute or chronic infection) and virally suppressed animals (Fig 1D). SIV RNA was also detected in all SIV-infected groups (P<0.05 for all; Fig 1E), albeit at significantly higher levels in the gut than in frontal cortex tissue, supporting a more active level of SIV transcription in the gut versus brain that persists despite ART. Interestingly, plasma viral load strongly correlated with vDNA+ cells (ρ = 0.52, P = 0.013; Fig 1F) and vRNA+ cells (ρ = 0.74, P<0.001; Fig 1G) in transverse colon, but not vRNA or vDNA+ cells in the frontal cortex of untreated animals, suggesting that the reservoir in frontal cortex tissue is less likely to be influenced by peripheral viral load (S1 Table). Together, these findings show that the frontal cortex is a stable reservoir of SIV despite ART-mediated viral suppression.

### SIV-infected animals have a higher frequency of myeloid cells in frontal cortex tissue than uninfected animals

Next, to determine the effect of SIV infection on immune activation in the brain, we first assessed the percentage of myeloid cells and astrocytes in brain tissue by spatial multiplex immunofluorescence imaging and quantitative analysis. GFAP+ cells were defined as reactive astrocytes, which are a key brain resident cells involved in brain structure, BBB formation, and neuronal maintenance via metabolic processing. Iba1+ cells were defined as myeloid cells (including microglia and perivascular macrophage), which play key roles as immune phagocytes in the brain. Untreated (acute and chronic) SIV+ animals and ART-suppressed animals had a higher frequency of Iba1+ myeloid cells in frontal cortex tissue relative to uninfected controls (Table 2; P<0.05 for all), consistent with an active immune response in the brain that is not resolved by ART. Due to known differences in both the cellular composition and function of grey and white matter of the brain (grey matter harbors neuronal cell bodies, relatively few microglia and is important in information processing, while white matter harbors myelinated neuronal axons and a large proportion of astrocytes), sub-analysis per region of frontal cortex tissue was performed.

**Table 2:** Cell phenotypes in the brain of SIV+ NHPs.

		SIV+			P value			
	SIV-	Acute	Chronic	VS	SIV- vs. Acute	SIV- vs. Chronic	SIV- vs. VS	Chronic vs. VS
Iba1	9.32 (8.18, 11.4)	18.9 (17.1, 20.0)	20.9 (15.7, 25.7)	20.0 (17.4, 28.7)	0.029	**0.004**	**0.008**	0.965
GFAP	17.7 (15.4, 20.6)	12.5 (11.2, 14.8)	14.0 (12.5, 17.7)	9.48 (7.71, 20.8)	0.057	0.170	0.177	0.091

ART: antiretroviral therapy, GFAP: glial fibrillary acidic protein, Iba1: ionized calcium binding adaptor molecule 1, NHP: nonhuman primate, SIV: simian immunodeficiency virus, VS: virally suppressed

Percentage of total cells (Median and interquartile range shown)

P value as determined by Mann Whitney U Test (P<0.05 statistically significant)

Bold values indicate significance (P<0.05), underlined values indicate trend (P>0.05, <0.1)

Elevated numbers of myeloid cells were present in both white and grey matter of SIV+ animals (untreated and ART-treated), indicating ongoing immune recruitment throughout regions of frontal cortex and not just in white matter alone, where microglia would be more commonly present (S1A Fig). Somewhat surprisingly, the percentages of GFAP+ reactive astrocytes were not significantly altered in SIV+ animals in frontal cortex tissue (Table 2), apart from a trend to lower percentage of GFAP+ cells in untreated SIV-infected animals relative to uninfected controls (P = 0.057; Table 2).

### SIV infection is associated with enhanced inflammatory type 1 IFN responses in frontal cortex brain tissue despite ART

**Fig 2:**
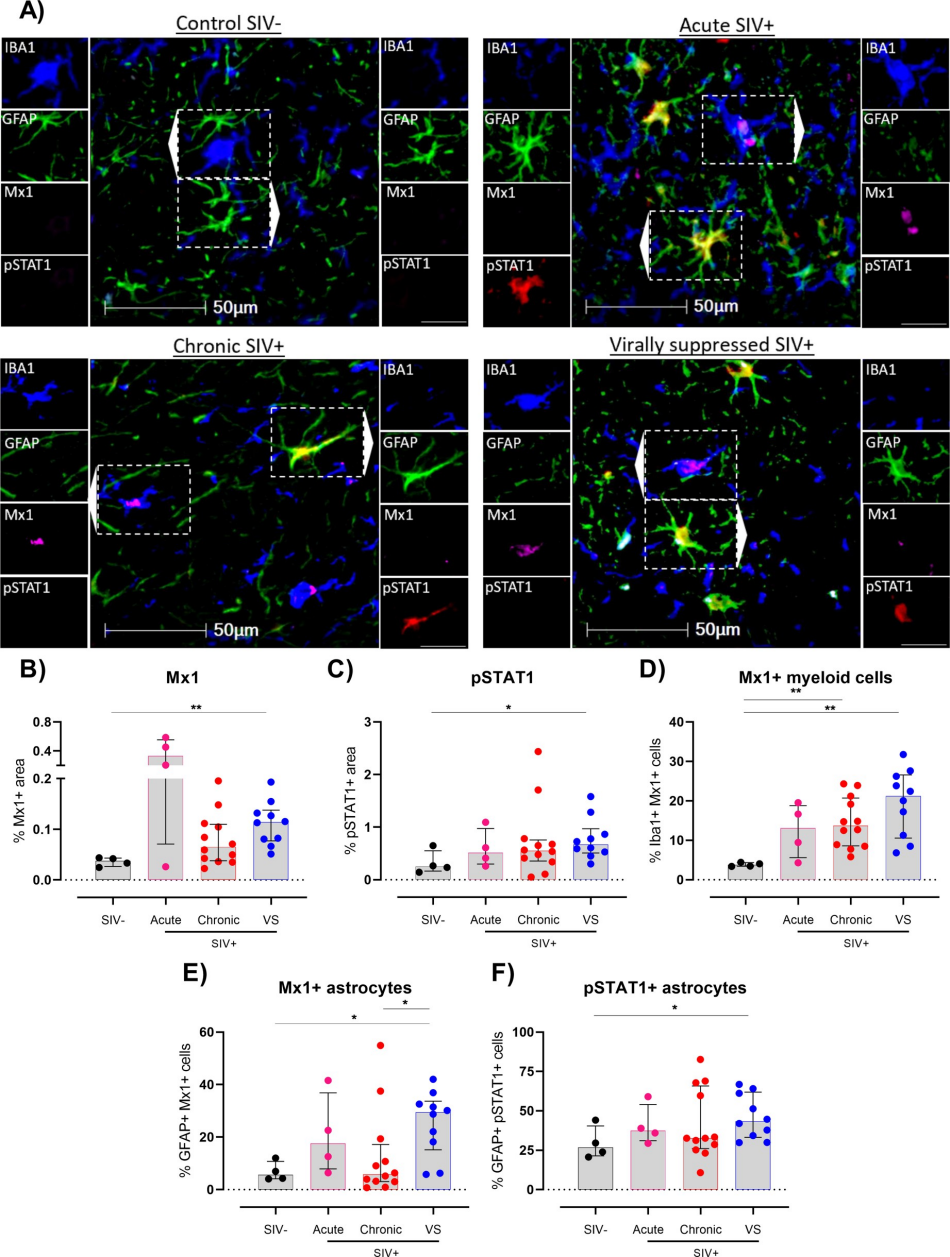
Chronically ART-suppressed SIV+ rhesus macaques show activated type I interferon responses in the frontal cortex of the brain. **(A)** Representative images of multiplex fluorescent immunohistochemistry performed on frontal cortex tissue of untreated, virally suppressed (VS) or uninfected controls for detection of Iba1 (myeloid cells), GFAP (astrocytes), pSTAT1 and Mx1. Images shown at x30 magnification; scale bars: 50μm and 25 μm (single channel inset). **(B)** Percentage of Mx1+ area, **(C)** pSTAT1 area, **(D)** Iba1+ Mx1+ cells as a percentage of total Iba1+ cells, **(E)** GFAP+ Mx1+ cells as a percentage of total GFAP+ cells or **(F)** GFAP+ pSTAT1+ cells as a percentage of total GFAP+ cells in frontal cortex brain tissue of viremic or virally suppressed (VS) SIV+ rhesus macaques. Median and interquartile ranges shown. All comparisons made using Mann-Whitney U test (*P<0.05, **P<0.01).

To further characterize the effects of both unsuppressed and ART-suppressed SIV infection on immune activation in frontal cortex brain tissue, we performed additional spatial multiplex immunofluorescence analysis using antibodies specific to cellular targets of inflammatory antiviral/type I interferon responses (e.g. Mx1, pSTAT1), anti-inflammatory/immune regulatory (e.g. TGF-β1, pSMAD3) and reactive oxygen species generation (e.g. SOD1) in combination with cell markers for Iba1+myeloid cells and/or GFAP+ astrocytes (representative images shown for each group; Fig 2A and S2 Table). SIV+ animals during acute infection had larger areas in frontal cortex tissue expressing the inflammatory/antiviral type 1 interferon induced GTP protein Mx1 relative to uninfected controls with a trend to higher levels in chronically infected animals (P = 0.078; Fig 2B). Surprisingly, levels of Mx1 were also elevated in virally suppressed animals (P<0.01; Fig 2B). Similarly, there were higher levels of phosphorylated STAT1 (pSTAT1), a transcription factor downstream of type I IFN receptor signaling, in virally suppressed animals (Fig 2C), suggestive of chronic activation of inflammatory type I interferon pathways in virally suppressed SIV infection.

To understand which brain cell types contributed to activated type I IFN responses, phenotypic analysis was performed for Iba1+myeloid cells and GFAP+ astrocytes. SIV+ animals with untreated chronic infection had a ~3.5-fold higher percentage of Iba1+ cells expressing Mx1 than uninfected controls (Fig 2D, P<0.05 for all). Importantly, virally suppressed SIV+ animals had an even higher frequency of myeloid cells expressing Mx1 (~20% of all Iba1+ cells vs ~4% for controls; Fig 2D), highlighting an expansion of activated myeloid cells in the frontal cortex during ART. Interestingly, sub-analysis by region showed that Mx1+ myeloid cells were preferentially elevated in grey matter of virally suppressed animals (S1B Fig), indicating an expansion of myeloid cells with activated type I interferon pathways in regions rich with neuronal cell bodies. Virally suppressed animals also showed high frequencies of astrocytes producing antiviral Mx1 (Fig 2E) or its upstream signaling factor pSTAT1 (Fig 2F) with the former almost 7-fold higher than uninfected controls (~30% vs ~4%). Together, these findings highlight that virally suppressed SIV+ animals display a chronically activated type I interferon signature in both myeloid cells and astrocytes in frontal cortex of the brain.

### SIV infection is associated with an increase in SOD1+ astrocytes in the frontal cortex tissue

**Fig 3:**
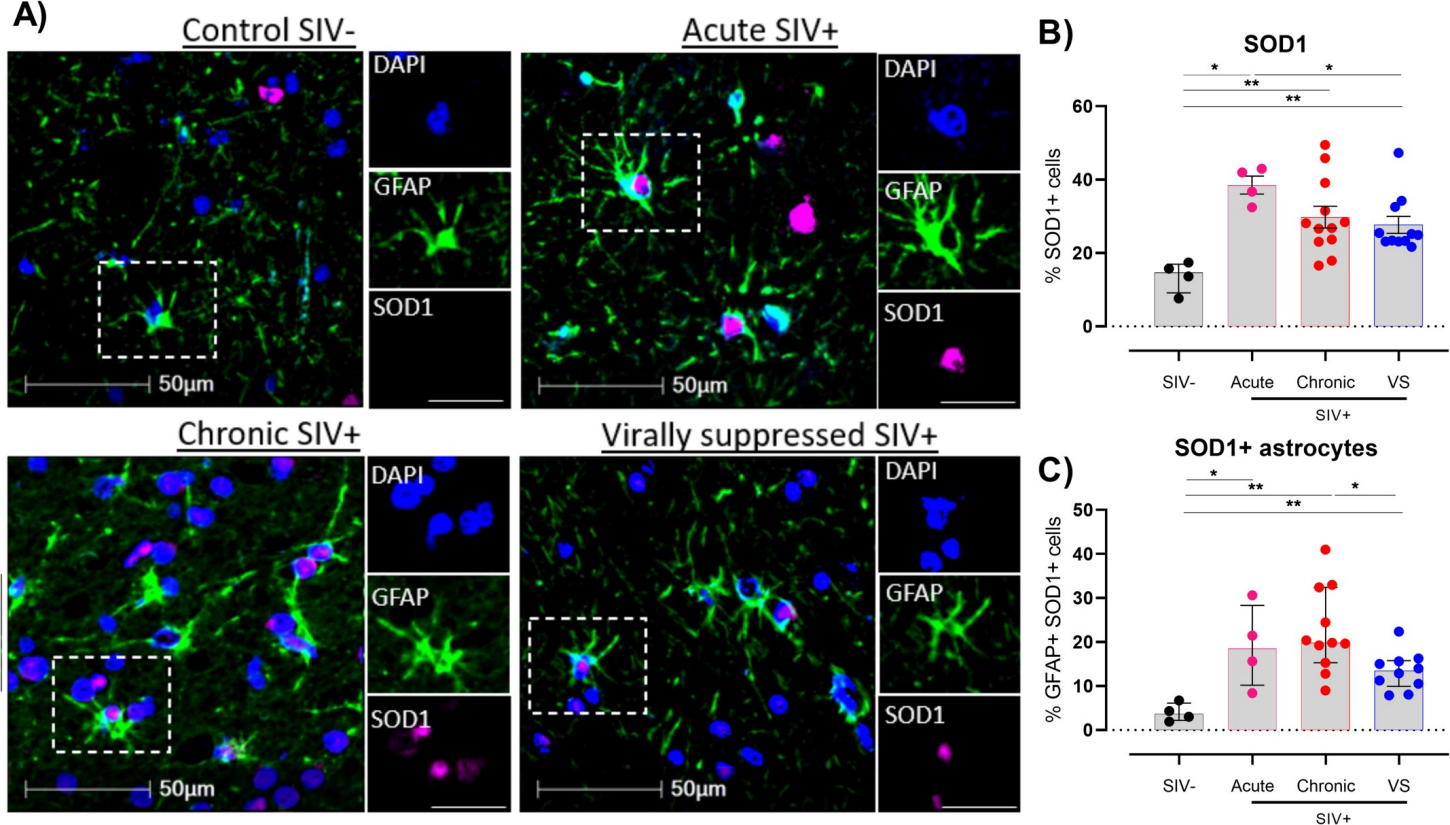
SIV infection enhances SOD1 production in the frontal cortex in the brain. **(A)** Representative images of frontal cortex tissue of SIV infected (acute, chronic or virally suppressed [VS]) or uninfected controls was stained using fluorescent multiplex immunohistochemistry to detect GFAP (astrocyte), SOD1 and DAPI (4′,6-diamidino-2-phenylindole). Images shown at x30 magnification; scale bars: 50μm and 25μm (single channel inset). **(B)** Frequency of total SOD1+ cells or **(C)** SOD1+ astrocytes in frontal tissue. Comparisons made using Mann-Whitney U test, *P<0.05, **P<0.01, ***P<0.001). Median and interquartile ranges shown.

We next sought to determine the effects of SIV infection on the generation of reactive oxygen species, which may contribute to localized neuroinflammation in the frontal cortex of the brain. The percentage of cells expressing superoxide dismutase 1 (SOD1), required for converting harmful superoxides to hydrogen peroxide, in frontal cortex tissue was ~2 fold higher than uninfected controls during acute SIV infection and remained elevated in chronically infected animals (Fig 3A–3C). Interestingly, levels remained elevated in virally suppressed SIV+ animals indicating that viral suppression with ART was unable to resolve this phenotype. Importantly, the percentage of SOD1+ astrocytes relative to total cells was dramatically elevated during acute infection (~20 fold) and remained elevated in both chronically infected and virally suppressed SIV+ animals (Fig 3C), indicating that astrocytes were major contributors to the heightened frequency of SOD1+ cells in the frontal cortex of these animals. Elevated levels of SOD1+ astrocytes were not just restricted to the white matter, as levels remained elevated in grey matter (S1C Fig), which may reflect a response to ongoing oxidative stress and potential direct damage to neurons. Limited expression of HIF-1α and IDO1 were observed regardless of infection status.

### SIV infection is associated with an expansion of TGF-β1+ myeloid cells in the frontal cortex

**Fig 4:**
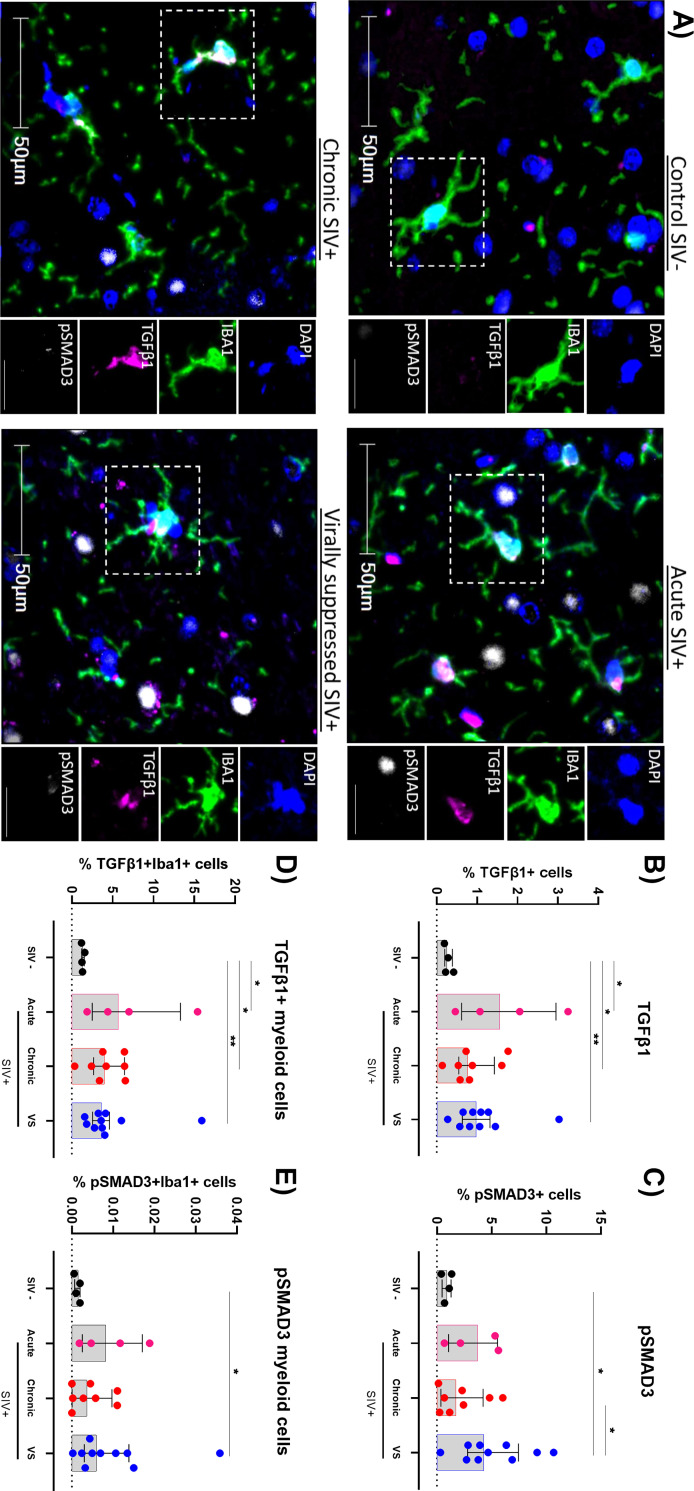
TGF-β1 pathways remain activated in SIV+ animals despite viral suppression with ART. **(A)** Representative images of IHC performed on frontal cortex tissue of SIV+ animals (acute [n = 4], chronic [n = 8] or virally suppressed [VS; n = 11]) or uninfected controls (n = 4) for Iba1 (myeloid cells), pSMAD3, TGF-β1 and DAPI. Images shown at x30 magnification; scale bars: 50μm and 25μm (inset). **(B)** Percentage of TGF-β1+ cells, **(C)** pSMAD3+ cells, **(D)** Iba1+ TGF-β1+ as a percentage of total Iba1 cells or **(E)** Iba1+ pSMAD3+ as a percentage of Iba1+ cells in frontal tissue matter. Median and interquartile ranges shown. Comparisons made using Mann-Whitney U test (*P<0.05, **P<0.01).

To understand whether SIV infection induced responses that counter heightened immune activation in the brain were observed (similar to what we have reported in peripheral tissues [42,43]), we performed additional spatial multiplex immunofluorescence analysis for TGF-β1 and phosphorylated SMAD3 (pSMAD3) within the frontal cortex (representative images shown; n = 4 chronic SIV+ animals excluded due to missing tissue sections; Fig 4A). Acute SIV infection was associated with a higher frequency of TGF-β1+ cells in frontal brain tissue relative to controls, which remained elevated in chronically infected animals (P<0.05 for both, Fig 4B). TGF-β1+ cells were also present at a higher frequency in virally suppressed animals than controls (P<0.05 for all comparisons; Fig 4B), consistent with a heightened state of immune activation. Similarly, the frequency of cells expressing pSMAD3, an important downstream TGF-β1 signaling protein, were elevated in virally suppressed animals (Fig 4C). Cells expressing TGF-β1+ were elevated during acute and chronic SIV infection and remained elevated in virally suppressed animals (Fig 4D; P<0.05 for all comparisons), suggesting that myeloid cells were major contributors to the expansion of total TGF-β1+ cells, in treated or untreated SIV infection in the frontal cortex.

The frequency of pSMAD3+ myeloid cells was higher in virally suppressed animals, albeit at low levels of detection (Fig 4E, P<0.05 for both), suggesting heightened flux via TGF-β1 signaling in animals on treatment. The expansion of TGF-β1+ myeloid cells was not restricted to white matter, as levels were also higher in the grey matter of virally suppressed SIV+ animals (S1D Fig), potentially in response to heightened immune activation as shown above. Together, frontal cortex tissue of ART-treated SIV+ animals showed a robust TGF-β1 signature, mainly associated with myeloid cells, which further supports active inflammatory and counter inflammatory immune responses in the brain of these animals.

### The blood-brain barrier is impaired in SIV+ animals on ART

**Fig 5:**
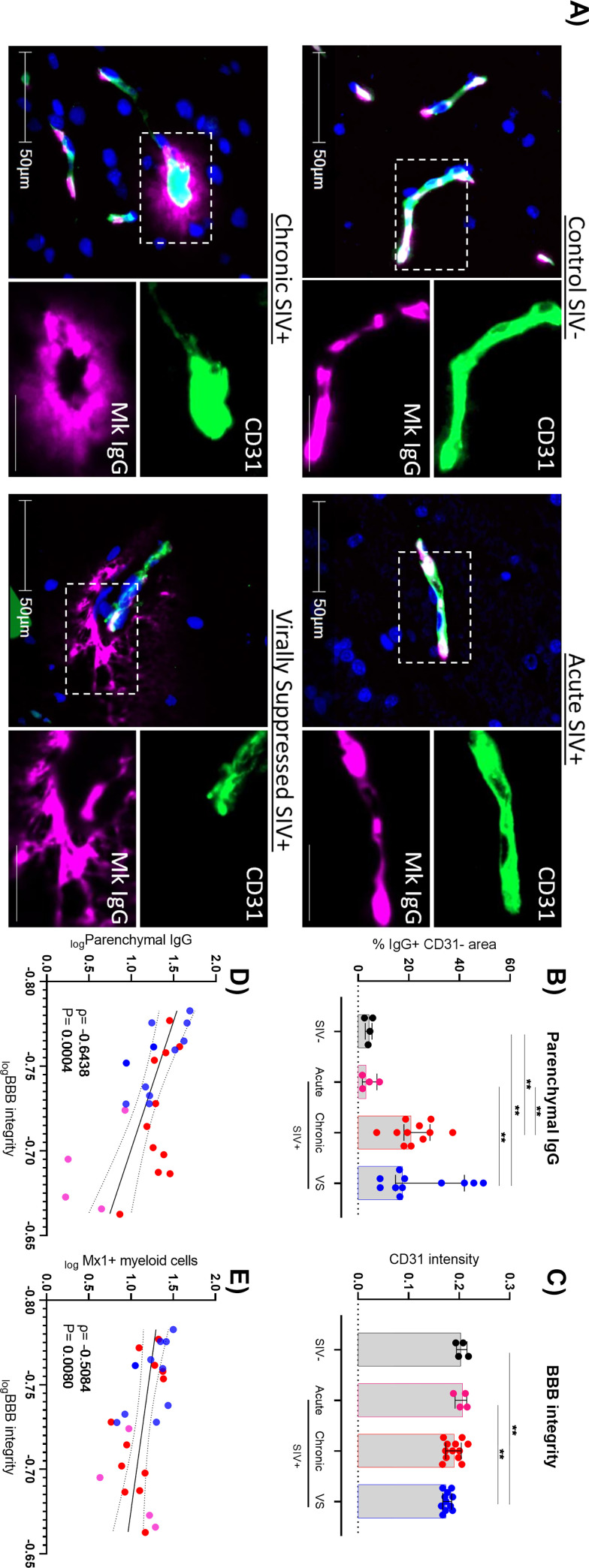
**The blood brain barrier (BBB) is impaired during ART-suppressed SIV-infection (A)** Representative images of multiplex immunohistochemistry labelling of CD31 (green) or IgG (pink) expression in frontal cortex. Scale bars: 50μm and 25μm (inset). **(B)** Frequency of parenchymal rhesus macaque (RM) IgG (IgG+ CD31-) or **(C)** fluorescence intensity of CD31 in brain tissue from either virally suppressed (n = 11; VS; blue) or untreated RM during acute (n = 4; pink) or chronic infection (n = 12; red) relative to uninfected controls (n = 4; black). Comparisons made using Mann-Whitney U test, **P<0.01. Median and interquartile ranges shown. **(D)** Correlative analysis of parenchymal IgG or **(E)** Mx1+ myeloid cells with BBB integrity in SIV infected or uninfected animals. Spearman rho and P value shown.

To determine whether heightened inflammation and immune activation with concomitant elevated immunoregulatory pathways in the frontal cortex of the brain were related to a breakdown of the BBB, BBB integrity was measured. BBB integrity was assessed by previously described methods via both the expression level (mean intensity) of immunofluorescent stained platelet endothelial cell adhesion molecule (PECAM; CD31) in frontal cortex tissues and by measuring the staining and diffusion of rhesus IgG in the parenchyma of the brain away from blood vessels (representative images shown in Fig 5A) [44,45]. Specifically, vascular expression of CD31 was associated with BBB stability [44]. Chronic and virally suppressed SIV+ animals showed 4-5-fold higher levels of parenchymal IgG in frontal cortex tissue than uninfected animals (Fig 5B), indicating breakdown of the BBB leading to leakage of vascular proteins into the brain parenchyma, which was consistent across both white and grey matter (S1E Fig). This was supported by lower levels of CD31 in virally suppressed animals, indicative of brain blood vessel endothelial cell changes that remain despite ART (Fig 5C). Interestingly, animals sacrificed during acute infection retained BBB integrity at levels similar to control animals, suggesting that effects on BBB integrity is progressive and manifested later in the disease course.

Importantly, levels of CD31 on brain vascular endothelial cells were inversely associated with levels of parenchymal IgG in the frontal cortex (Fig 5D, ρ = -0.644, P<0.001), directly demonstrating breakdown of the BBB and the passaging of vascular proteins into the brain of ART-treated macaques. Furthermore, measures of BBB integrity was inversely associated with levels of Mx1+ myeloid cells (ρ = -0.508, P = 0.008; Fig 5E), highlighting that BBB disruption was associated with heightened immune activation/inflammation within the frontal cortex of SIV+ animals.

**Fig 6:**
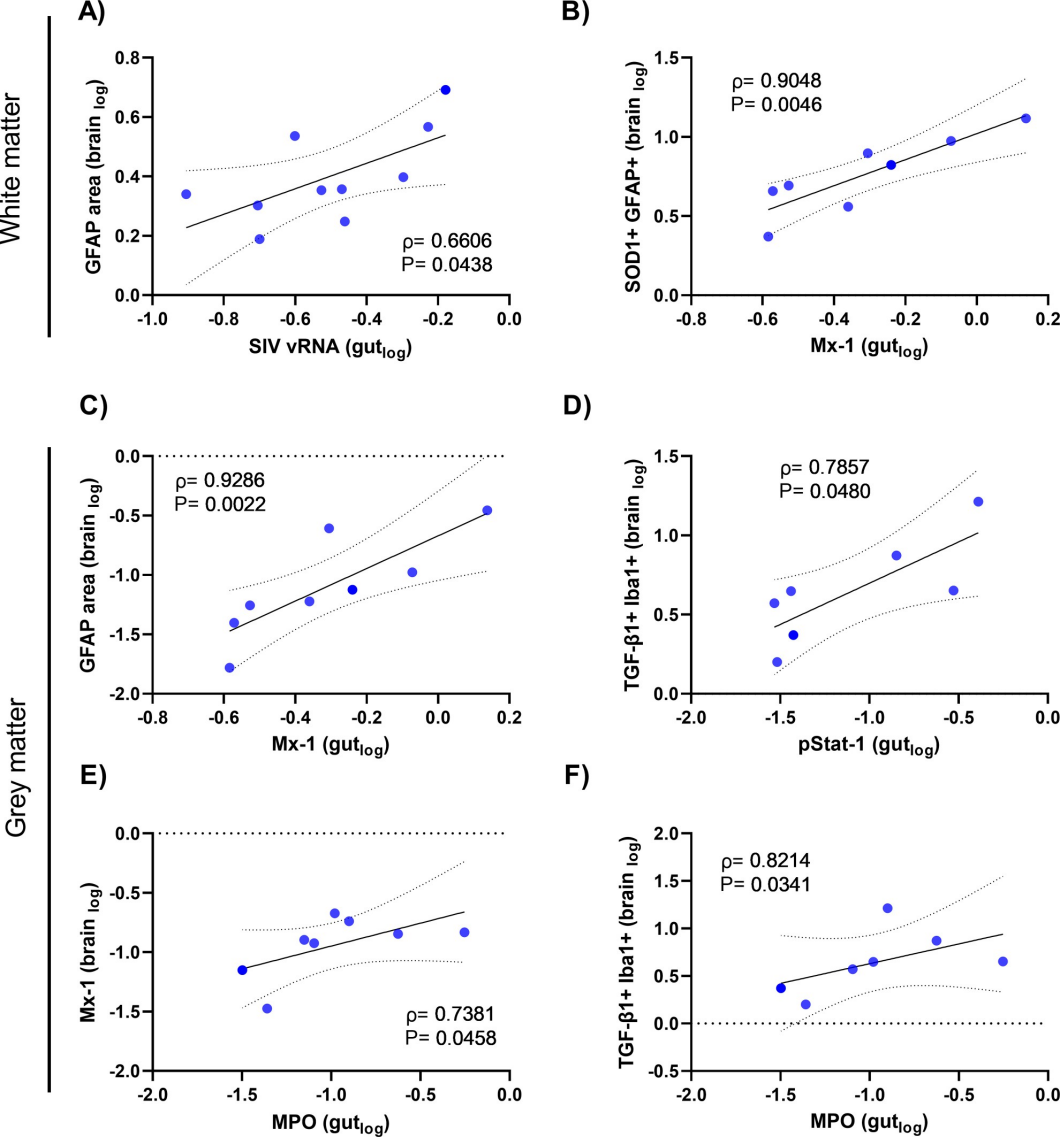
SIV RNA, type I interferon responses and barrier damage in the gut are associated with immune activation in the frontal cortex of virally suppressed SIV+ animals. Correlative analysis of levels of immune activation markers in white matter (WM) or grey matter (GM) from frontal cortex tissue of virally suppressed SIV+ animals (n = 7–10), as determined by IHC, to levels of **(A)** SIV viral RNA, **(B and C)** Mx1, **(D)** pSTAT1 or **(E and F)** myeloperoxidase (MPO)+ cells from matched gut tissue. Parameters were log transformed. Spearman rho and P values shown. P<0.05 considered statistically significant.

SIV viral persistence and immune activation in the gut is associated with immune activation in the frontal cortex of ART-treated NHPs. To understand whether neuroinflammation and immune activation in the frontal cortex was directly associated with viral persistence in the brain (SIV RNA+ or DNA+ cells), we performed a correlation analysis on virally suppressed SIV+ animals. We reasoned that if viral persistence in the brain was driving neuroinflammation that the number of cells actively expressing SIV (i.e. SIV RNA+, but not latently infected cells (i.e. SIV DNA+) would correlate with the magnitude of neuroinflammation and immune activation. Conversely, markers of neuroinflammation remained elevated at similar levels in SIV+ animals either with or without detectable SIV RNA present in brain frontal cortex tissue (S2 Fig). Furthermore, limited overt associations between SIV vRNA/vDNA+ cells in frontal cortex tissue and markers of neuroinflammation were observed (S3 Table and S3 Fig). Instead, SIV RNA+ cells and measures of immune activation/inflammation in matched gut tissues (assessed in a similar fashion to the brain tissue) correlated with a range of inflammatory processes in frontal cortex tissue in virally suppressed SIV+ animals (Fig 6).

Active SIV transcription in the gut (defined as productively infected vRNA+ cells with a classic “starburst” staining pattern with vRNA signal encompassing both the cell nucleus and cytoplasm) was associated with astrocyte activation (defined by the area of GFAP staining) within the frontal cortex of the brain, supporting a link between active SIV infection in the gut and astrocyte activation in the brain (ρ = 0.661; P = 0.044; Fig 6A). Cells expressing type I IFN responsive gene product Mx1 in the gut of virally suppressed SIV+ animals also correlated with a greater level of activated astrocytes (SOD1+ GFAP+ cells) in white matter (ρ = 0.905; P = 0.005; Fig 6B). Levels of Mx1+ cells in the gut were also strongly associated with astrocyte activation in the grey matter (ρ = 0.929; P = 0.002; Fig 6C), indicating that these effects were not localized to the white matter alone. Moreover, interferon receptor signaling in the gut (pSTAT1+ cells) strongly correlated with myeloid cells expressing regulatory TGF-β1+ in grey matter (ρ = 0.786; P = 0.048; Fig 6D). Finally, measures of impaired gut barrier integrity (as defined by numbers of infiltrating neutrophils; myeloperoxidase [MPO]+ cells) strongly correlated with total Mx1 levels (ρ = 0.738; P = 0.046; Fig 6E) and TGF-β in grey matter (ρ = 0.821; P = 0.034; Fig 6F), implicating gut damage as a potential contributor to cellular activation in the frontal cortex of the brain of ART-suppressed animals.

### Gut damage alone can induce neuroimmune activation/inflammation independent of SIV infection

As our findings in SIV+ animals showed a link between both SIV RNA and local inflammation in the gut with neuroinflammation in frontal cortex tissue, we next sought to understand whether gut damage alone was sufficient to induce inflammatory and immune activation pathways in the brain. Chronic gut damage and impaired barrier function is a hallmark of SIV/HIV infection that is not resolved by ART. Therefore, cellular activation in frontal cortex tissue was measured in our well validated NHP model of chronic colitis, which recapitulates the extent and effects of gut damage seen in SIV+ animals [34]. SIV uninfected animals were treated with six cycles of DSS in their drinking water (1 cycle = 14 days DSS followed by 14 days normal water) to generate chronic colitis prior to sacrifice and tissue collection. As expected [34], DSS-treated animals had impaired gut integrity, as defined by significant epithelial breaches and higher levels of infiltrating neutrophils in the gut (S4A Fig, P<0.05).

**Fig 7:**
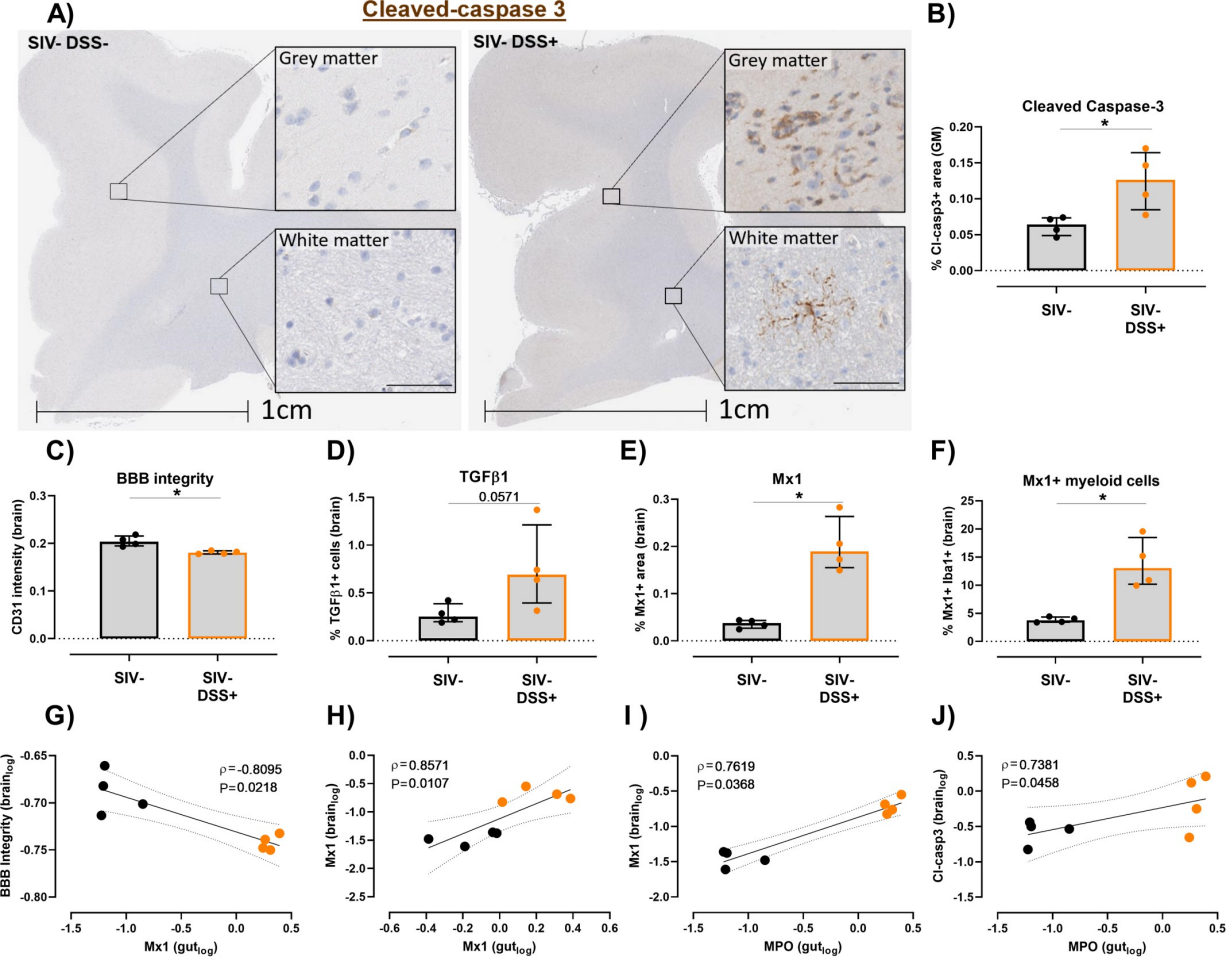
Gut damage induces cellular activation in the frontal cortex of the brain in the absence of viral infection. **(A)** Representative images of cleaved caspase-3 (Cl-casp3) staining in the grey and white matter of the frontal cortex of SIV uninfected (black; n = 4) or SIV-uninfected DSS-treated (SIV-DSS+; orange; n = 4) rhesus macaques. Scale bars: 1 cm (overview) and 50 μm (insert). **(B)** Percentage area of Cl-casp3+ tissue in grey matter. Comparison of levels of **(C)** blood brain barrier (BBB) integrity (CD31 intensity), **(D)** TGF-β1, **(E)** Mx1 or **(F)** % Iba1+ Mx1+ as a percentage of Iba1+ cells between DSS untreated (n = 4) and treated animals (n = 4). Comparisons made using Mann-Whitney U test, median and interquartile ranges shown (*P<0.05). **(G-J)** Correlation of immune activation markers in gut and brain tissue. Spearman Rho and P values shown.

Larger regions of inflammation, as measured by expression of Mx1 or pSTAT1 in the colon, was also observed indicative of chronic colitis in the gut (S4C and S4D Fig, P<0.05 for all). Similar to our previous findings [34], DSS-treated animals had higher levels of IL-8, CCL11, IL-12p70 and IL-6 in plasma compared to pre-treatment conditions (S4E Fig), supportive of systemic inflammation. Importantly, SIV-uninfected DSS-treated animals also showed heightened immune activation and inflammation in frontal cortex tissue. DSS-treated animals had a higher frequency of apoptotic cells expressing cleaved-caspase 3 (Cl-casp3) in grey matter than untreated animals (P<0.05, Fig 7A and 7B), indicative of cell death in the frontal cortex independent of viral infection. Similar to ART-treated SIV-infection, SIV uninfected DSS+ animals showed breakdown of the BBB (Fig 7C) and heightened levels of immune regulatory TGF-β1+ cells (P = 0.057; Fig 7D). The frequency of total Mx1+ regions (Fig 7E) and the frequency of myeloid cells expressing Mx1 were elevated relative to untreated animals (Fig 7F).

Correlative analysis demonstrated that the frequency of inflammatory Mx1-expressing cells in the gut was associated with BBB breakdown (ρ = -0.81; P = 0.022; Fig 7G). Levels of Mx1 in the gut was positively associated with Mx1 levels in frontal cortex tissue (ρ = 0.86; P = 0.011; Fig 7H), indicating a direct association between immune activation in the gut and brain. Similar to findings in SIV+ animals, impaired gut integrity (defined by levels of infiltrating neutrophils, MPO+) was associated with inflammation (ρ = 0.76; P = 0.037; Fig 7I) and cell death in the frontal cortex (ρ = 0.74; P = 0.046; Fig 7J). Together, these data show that gut damage, related barrier dysfunction and resulting systemic inflammation and immune activation is strongly associated with BBB breakdown and changes in the neuroinflammatory profile of NHPs that are similar to those seen during long-term virally suppressed SIV-infection.

## Discussion

Here we provide the most comprehensive spatial analyses of both SIV persistence and related immune activation/inflammation in the frontal cortex of the brain in ART-suppressed SIV+ RMs. SIV vDNA (and vRNA for a subset of animals) were detected in the brain during acute infection and remained present in chronic infection, which was not reduced by viral suppression with ART. Importantly, frontal cortex tissue of ART-suppressed SIV+ animals remained in a chronically activated state that was characterized by BBB dysfunction and activated type I interferon responses in myeloid cells and astrocytes. Immune activation/inflammation in the frontal cortex of the brain was associated with SIV vRNA, barrier damage and immune activation/inflammation in the gut, supporting a role for systemic chronic inflammation related to gut damage (and other sources) as a contributor to neuroinflammation. Finally, using a NHP model of chronic colitis that recapitulates SIV-induced gut damage [34], we provide evidence to support that gut damage alone can contribute to immune activation/inflammation in the frontal cortex of the brain in a similar fashion to neuroinflammation in SIV+ animals. These findings indicate that chronic gut damage caused during SIV/HIV infection may be a significant contributor of neuroimmune activation/inflammation in PWH.

Our findings of elevated levels of Iba1+ myeloid cells, Mx1+ cells and SOD1+ astrocytes in frontal cortex tissue of ART-suppressed animals support a state of chronic inflammation and immune activation in the brain that may contribute to neurodegradation in PWH. Type I interferon responses, including Mx1 and pSTAT1 expression, have been associated with neuroinflammation and cognitive deficits in untreated SIV-infection and PWH [46,47]. Similar to our findings for SOD1, elevated SOD2 levels have been found in brain tissue of SIV+ animals [48], which together may reflect a response that counteracts ongoing oxidative stress. Interestingly, in contrast to previous findings for SOD2, levels of SOD1 remained elevated despite ART in this study suggesting that elevated cytosolic SOD1 persists despite therapy. No difference in HIF1α levels between groups was found in this study, which may reflect active oxygen stress pathways in these animals independent of hypoxia. Therefore, expanded analysis of oxidative stress pathways in the brain of ART-treated animals may be of interest.

We also found an accumulation of Iba1+ myeloid cells in the frontal cortex of virally suppressed SIV+ animals, which supports previous findings in humans of aberrant myeloid cell function in PWH [49,50]. Microglia perform important roles in immune maintenance in the brain, including phagocytosis of cellular debris, production of cytokines including TNF-α and IFN-γ, and are mainly found in white matter of healthy individuals. Thus, an expansion of these cells, especially in the grey matter, which harbors neuronal cell bodies and is important in information processing, may represent ongoing immune activation that can have adverse effects on neuronal health. Higher levels of TGF-β1 expression in myeloid cells in virally suppressed SIV+ animals were observed, indicating hyperactivated regulatory responses in the frontal cortex presumably in response to inflammation and immune activation. Although TGF-β1 is traditionally characterized as an anti-inflammatory cytokine, elevated CSF levels of TGF-β1 are present in people with Alzheimer’s disease [51] and in PWH [52,53], supporting a possible negative effect of excessive levels in the brain.

Importantly, SIV+ animals had significant breakdown of the BBB, which was not restored by ART. The BBB regulates recruitment of immune cells and the passive transfer of potentially toxic substances into the brain, and BBB breakdown has been associated with severe cognitive impairment [54]. Thus, BBB dysfunction in these animals may facilitate the transport of inflammatory cytokines, or recruitment of other immune cells into the CNS, which likely accounts for heightened measures of inflammation and immune activation in the brain. These findings differ slightly from those in humans where ~30% of humans show BBB breakdown during acute infection [55] and long-term ART use improves BBB integrity in PWH [54], both of which were not observed in SIV-infected macaques in this study. This may be due to our assessment of the BBB barrier itself via immunofluorescence-based staining for endothelial integrity and IgG penetration into the brain parenchyma instead of the measurement of surrogate plasma/CSF biomarkers (i.e. albumin levels).

SIV vDNA+ cells were present in the frontal cortex of the brain of ART-suppressed SIV+ animals, confirming that the brain is a stable reservoir of SIV, as we and others have shown in humans and SIV+ NHPs [5,12,23,26,56]. Importantly, the size of the SIV reservoir remained stable between chronically viremic and ART-suppressed SIV+ animals, highlighting that suppression of SIV viremia in the periphery has little effect on the brain reservoir. SIV vRNA was not detected in the frontal cortex of most virally suppressed SIV+ animals (7/11; Fig 1), suggesting that ART treatment is able to effectively reduce the active viral reservoir, without reducing the total reservoir size in this tissue. In this study SIV persistence (vDNA or RNA) in the frontal cortex did not overtly associate with widespread changes in neuroimmune activation/inflammation. However, it is possible (and likely) that local immune activation and inflammation may occur in local cellular neighbourhoods surrounding SIV (and by extension HIV) vDNA or vRNA+ cells in the brain. Expanded studies in larger cohorts of non-human primates and human tissues are required to dissect the association of viral DNA, RNA and intact potentially replication competent viruses and local immune activation in the brain.

Our findings that heightened immune activation in the gut are strongly associated with markers of neuroinflammation (even in the absence of SIV) provides evidence linking gut damage, immune activation and neuroinflammation. These data support and extend previous studies demonstrating associations between plasma/CSF measures of gut-associated inflammation/immune activation (including plasma IL-6, sCD14, neopterin, cellular HIV reservoirs), monocyte transmigration and surrogate measures of brain damage and cognitive impairment in humans and animals [57–60]. Furthermore, an unblinded single-arm clinical trial targeting gut-associated immune activation using cenicriviroc (a dual CCR2 and CCR5 antagonist) has also shown preliminary evidence of improvement in learning-based measures of cognitive performance in HIV [61]. However, further extensive studies demonstrating beneficial changes across multiple cognitive domains are required to determine whether targeting systemic inflammation is an effective method of improving neuroinflammation and cognitive status in PWH. We and others have previously shown that gut damage, microbial translocation, and systemic inflammation are hallmarks of SIV/HIV infection that is not fully restored during suppressive ART treatment [30,62,63]. Therefore, chronic gut damage and immune activation in ART-suppressed SIV/HIV infection may, in part, be a significant contributor to neuroinflammation that cannot be resolved by ART treatment alone.

Importantly, our findings of heightened measures of neuroinflammation in SIV-uninfected DSS-treated animals provides further evidence in NHPs that GI tract damage alone can contribute to neuroimmune activation/inflammation, even in the absence of SIV infection. Gut damage induced by DSS-treatment facilitates immune activation in the gut including neutrophil recruitment, T cell activation and type I interferon production [34]. We have also previously shown that DSS-treatment facilitates translocation of microbial products into the lamina propria of the colon and systemically, including in axillary lymph nodes, and leads to heightened levels of IL-8 in plasma (also observed in this study; S4E Fig). This supports ongoing microbial translocation as a source of systemic inflammation and immune activation in these animals, which may contribute to BBB dysfunction and neuroimmune activation [34]. However, larger prospective studies are required to define the discrete mechanisms by which gut damage and/or systemic inflammation can induce neuroinflammation.

It is important to consider that other mechanisms including coinfection with other endemic pathogens including rhesus cytomegalovirus (CMV) or endogenous retroviruses may also contribute to gut inflammation and/or neuroinflammation in these animals either via localized reactivation or the induction of systemic inflammation [64]. Animals in this study were pre-screened for macacine herpes virus 1 (herpes B), SIV, simian T-lymphotropic virus and simian retrovirus, but not rhCMV, which is assumed to be endemic in rhesus macaques by adolescence. In this study all DSS-treated SIV-uninfected animals did not have CMV reactivation present in brain tissue as tested by RhCMV immunohistochemistry staining. However, as hCMV is strongly associated with altered immune function and phenotypes and is almost ubiquitously present in PWH, further studies into the impact of these pathogens within distinct tissue microenvironments will be important.

Together, further extensive studies are required to define the precise mechanisms by which gut damage in SIV-infection contributes to neuroinflammation and possible cognitive impairment. Moreover determining the effect of gut damage either independently or in concert with other potential cofounding factors including coinfection, and traditional risk factors including smoking, drug use etc on neuroinflammation is essential in the context of PWH.

These findings also have important implications for other neurological disorders where measures of chronic low-level inflammation, potentially driven by the gut-brain axis or infections, are associated with disease pathogenesis including Alzheimer’s disease, multiple sclerosis, and Parkinson’s disease [65–67]. Understanding the links between gut damage and brain health may offer critical insight into improving cognitive outcomes in PWH on suppressive ART.

There are several considerations that may impact the outcomes of this study. Frontal cortex/cerebrum was assessed as HAND is considered to mainly affect this region of the brain and HIV viral DNA is present at higher levels than other parts of the brain. However, it would be of interest to measure both viral persistence and immune activation/inflammation in multiple regions of the brain. Furthermore, assessment of the cognitive status of animals would be of interest, although this is difficult to perform due to a lack of standardised protocols. The use of a cross-sectional cohort may have contributed to greater variability in groups, particularly animals with acute infection. Expansion of these studies in larger, dedicated cohorts, potentially also examining the effect of the gut microbiome in animals and/or adjunctive anti-inflammatory treatments have on brain inflammation, would be of interest.

In summary, we comprehensively define the viral and inflammatory dynamics in the frontal cortex of the brain during both viremic and virally suppressed SIV infection in NHPs. Our observations of persistent viral reservoirs and heightened measures of immune activation/inflammation in frontal cortex tissue are associated with gut inflammation, highlight the potential pathways for ongoing neurocognitive impairment in PWH. Thus, further defining how gut damage may contribute, and/or exacerbate these effects may lead to better health outcomes for PWH.

## Materials and methods

### Ethics statement

All animals were housed and treated in accordance with Association for the Assessment and Accreditation of Laboratory Animal Care (AAALAC) standards and following ethics approval by each institution; namely the Institutional Animal Care and Use Committee (IACUC) of the National Cancer Institute, the Yerkes National Primate Research Center, Emory University and Advanced Bioscience Laboratories, as previously described [26,34–37].

### Nonhuman primate cohort

This study was performed with a retrospective cohort of 16 non-virally suppressed SIV+ (4–68 WPI) RMs (*Macaca mulatta*), 11 ART-suppressed SIV+ (36–68 WPI) RMs and 8 SIV-uninfected RMs (Table 1). RMs were infected intravenously with SIV_mac239_, or the closely related SIV_mac251_. Brain tissue was collected immediately post-mortem and fixed in paraformaldehyde.

### In situ SIV DNA and RNA analysis and quantification

The detection of SIV RNA and DNA on brain and colon tissue from the RM cohort were completed using RNAscope, as described [35]. Whole tissue sections were analyzed separately according to grey and white matter for brain samples, and gut-associated lymphoid tissue (GALT) and lamina propria (LP) for colon samples. Sections were analyzed for the total number of vRNA+ and vDNA+ per 10^5^ total cells using HALO software (v3.0.311.405; Indica Labs) ISH module (v3.0.3). Sections underwent blinded manual curation to correct false positives.

### Immunohistochemistry

Single chromogenic IHC was completed as described [68]. Briefly, heat induced epitope retrieval (HIER) was performed using 1x target retrieval buffer (322000; ACD), and antibody was detected with a 2-hour incubation in rabbit anti-human cl-casp3 (1:200; 9664; Cell Signaling) at RT. Whole tissue sections were analyzed separately according to grey and white matter for brain samples. The percentage of cl-casp3 area was calculated using HALO software (Area quantification v2.1.3; Indica Labs).

### IF assays

To assess cellular activation, multiplex IF assays were performed using antibody panels on consecutively sectioned and mounted frontal cortex and matched colon tissues (Panels/conditions listed in S2 Table).

Slides prepared for antibody detection as described previously [68], with HEIR completed at 110°C for 15 minutes in either Tris (pH9, DAKO) or Citrate (pH 6, Thermo) buffer. The first primary antibody was added to each slide and incubated for 1 hour or overnight at RT and was detected with an anti-rabbit or anti-mouse polymer horseradish peroxidase (HRP)- conjugated system (GBI Labs). TSA reagent conjugated with Alexa Fluor (Invitrogen) were used to visualize the corresponding antigen. To remove residual antibody for the next round of antigen detection, each slide was boiled for 15 minutes in citraconic anhydride (CA; 0.01% containing 0.05 tween20), Citrate pH6, DIVA (Biocare) or Tris pH9 retrieval buffer and left to cool at RT. This method was then repeated 2–3 times to complete each IF assay, excluding the BBB assay. RM IgG staining was performed following the above protocol. However, to allow fluorophore intensity analysis, anti-sheep AF488-conjugated secondary antibody (A-11015, Invitrogen; 3 hours) was used to detect CD31. Additionally, BBB integrity panel underwent a 45 minute photobleaching pre-treatment step in a fluorophore bleaching solution (4.5% (wt/vol) H _2_O_2_ and 20mM NaOH in PBS) after the first antigen retrieval, as described [69]. Nuclei was visualized with DAPI (Invitrogen) 1:10,000 for 10 minutes, washed in H_2_O and mounted with ProLong Gold Antifade Mountant (P36930; Invitrogen) and scanned (AxioScan; 20x magnification; Zeiss). Whole tissue sections were analyzed separately according to grey and white matter for brain samples, and GALT and LP for colon samples. IF was quantified using HALO software (Indica Labs) to establish the percentage of positive cells (HiPlex FL v4.0.4), percentage of positive area and the colocalization of antibodies (Object colocalization FL v1.0). Additionally, CD31 protein expression was measured using the mean fluorescence intensity of CD31+ objects.

### Plasma cytokine measurements

Plasma samples were analyzed in duplicate for IL-8, CCL11/Eotaxin, CXCL10/IP-10/CRG-2, CXCL11/I-TAC, IFN-gamma, IL-12p70, IL-2, IL-6, RANTES, TNF-alpha, and VEGF using a custom Luminex panel (R&D Systems NHP XL Cytokine Panel, Minneapolis, MN) following the manufacturer’s instructions. Analysis was completed using Milliplex LX-200 Analyzer (EMD Millipore, Billerica, MA) bead sorter with XPonent Software version 3.1 (Luminex, Austin, TX).

### Statistical analysis

Comparisons between groups were made using non-parametric Mann-Whitney U tests; medians and interquartile ranges shown. Spearman correlations were performed on log transformed data; rho and P values shown. P<0.05 considered significant. Analyses performed using GraphPad Prism software (version 9.2.0 Windows, GraphPad Software, La Jolla California USA).

## Supporting information

S1 Table Association of SIV RNA+ and DNA+ cells in the brain and peripheral sites in of SIV+ animals. (DOCX)

S2 Table Multiplex fluorescent immunohistochemistry. (DOCX)

S3 Table Association of SIV-infected cells and immune activation in the CNS. (DOCX)

S1 Fig Immune activation and blood brain barrier dysfunction in white and grey matter of SIV+ animals. Quantification of **(A)** myeloid cells (Iba1+), **(B)** Mx1+ myeloid cells, **(C)** SOD1+ astrocytes, **(D)** TGF-β1+ myeloid cells or **(E)** parenchymal IgG in grey (left panel) or white (right panel) of acute (pink), chronic (red) or virally suppressed (VS) SIV infection or uninfected controls (SIV-). Comparisons made using Mann-Whitney U tests. Median and interquartile ranges shown. *P<0.05; **P<0.01, ***P<0.001.
(TIF)

S2 Fig Immune activation in the frontal cortex of rhesus macaques stratified by SIV RNA+ and SIV RNA- cells in the brain. Quantification of **(A)** Iba1+ myeloid cells, **(B)** Mx1+ area, **(C)** % SOD1+ cells, **(D)** %TGF-β1+ cells, **(E)** IgG+CD31- area and **(F)** blood brain barrier (BBB) integrity in the frontal cortex of viremic or virally suppressed SIV+ animals expressing RNA+ (purple) or RNA- (green) in the brain. Comparisons made using Mann-Whitney U tests. Median and interquartile ranges shown. *P<0.05; **P<0.01, ***P<0.001.
(TIF)

S3 Fig Correlative analysis of SIV vDNA and RNA+ cells and measures of immune activation and inflammation in frontal cortex tissue of SIV+ animals. Correlative analysis of SIV vRNA or DNA+ cells and levels of immune activation (Mx1+ area or SOD1+ cells) or blood brain barrier dysfunction (parenchymal IgG+ area) quantified by multiplex immunofluorescence and DNA/RNAscope in frontal cortex tissue of virally suppressed (upper panels; blue; n = 10–11) or chronically SIV-infected rhesus macaques (lower panels; red; n = 11–12). All data log transformed. Samples with undetectable SIV RNA or DNA denoted -1 log vRNA+ (or DNA, as appropriate) cells/10^5^. Non-parametric Spearman’s rho (ρ) and P values shown. P<0.05 considered statistically significant.
(TIF)

S4 Fig Chronic DSS treatment induces gut damage, local immune activation, and systemic inflammation in SIV-uninfected rhesus macaques. **(A)** Representative multiplex immunofluorescence images of gut immune activation from SIV-uninfected rhesus macaques either treated with dextran sodium sulfate (DSS; right panel) or left untreated (left panel). Tissue labelled with CD68/CD163 (blue), myeloperoxidase (MPO; green), pSTAT1 (magenta), Mx1 (red) or Ki67 (grey). Scale bars 100 μm. Quantification of the frequency of **(B)** MPO, **(C)** Mx1 or **(D)** pSTAT1 expressing cells between DSS treated (n = 4) or untreated (n = 4) animals. Comparisons made using non-parametric Mann-Whitney U tests, median and interquartile ranges shown. **(E)** Longitudinal quantification of IL-8, CCL11, IL-12p70 and IL-6 in the plasma of SIV-uninfected rhesus macaques pre- and post-DSS treatment (n = 4) by Luminex immunoassay. Percent change from untreated controls shown. * P<0.05.
(TIF)
