# Supplementary material for: Chronic immune activation and gut barrier dysfunction is associated with neuroinflammation in ART-suppressed SIV+ rhesus macaques
Source: PLoS Pathog. 2023 Mar 29;19(3):e1011290. doi: 10.1371/journal.ppat.1011290 (PMC10085024; doi:10.1371/journal.ppat.1011290)
Supplement: S1 Table — (DOCX) [file ppat.1011290.s001.docx]

| **S1 Table. Association of SIV RNA+ and DNA+ cells in the brain and peripheral sites in SIV+ animals** | | | |
| --- | --- | --- | --- |
| **Parameter** | **vDNA gut** | **vRNA gut** | **pVL** |
| vDNA GM | ρ=0.230, P=0.302, | ρ=0.140, P=0.532 | ρ=0.046, P=0.819 |
| vDNA WM | ρ=-0.014, P=0.949 | ρ=0.027, P=0.905 | ρ=-0.103, P=0.608 |
| vRNA GM | ρ=0.342, P=0.118 | ρ=0.250, P=0.261 | ρ=0.252, P=0.205 |
| vRNA WM | ρ=0.377, P=0.083 | ρ=0.302, P=0.171 | ρ=0.244, P=0.220 |
| GM: grey matter; pVL: plasma viral load; vDNA: SIV viral DNA; vRNA: SIV viral RNA; WM: white matter  ^a^P value and rho determined by non-parametric Spearman correlation (P<0.05 statistically significant); n=27  Underlined values indicate trend (P<0.1) | | | |
