# Supplementary material for: Chronic immune activation and gut barrier dysfunction is associated with neuroinflammation in ART-suppressed SIV+ rhesus macaques
Source: PLoS Pathog. 2023 Mar 29;19(3):e1011290. doi: 10.1371/journal.ppat.1011290 (PMC10085024; doi:10.1371/journal.ppat.1011290)
Supplement: S2 Table — (DOCX) [file ppat.1011290.s002.docx]

| **S2 Table. Multiplex fluorescent immunohistochemistry** | | | | | | |
| --- | --- | --- | --- | --- | --- | --- |
|  | **Ab** | **Clone** | **Company** | **AR** | **Dilution** | **Detection** |
| Proinflammatory- CNS | GFAP | Rbt pAB | Sigma-Aldrich (#HPA056030) | Tris pH9 | 1:500 (1 hr) | TSA AF488 |
|  | Mx1 | Ms mAB (CL143) | EMD Millipore (#MABF938) | Citrate pH6 | 1:750 (1 hr) | TSA AF568 |
|  | pSTAT1 (Tyr701) | Rbt mAB (58D6) | Cell signaling (#9167S) | Tris pH9 | 1:200 (o/n) | TSA CF750 (1:1500) |
|  | Ki67 | Ms mAB (B56) | BD Pharmingen (#550609) | Citrate pH6 | 1:200 (1 hr) | TSA AF647 |
|  | Iba1 | Rbt pAB | Biocare Medical (#CP290A) | Citrate pH6 | 1:500 (o/n) | TSA AF350 (1:50) |
| Proinflammatory- Gut | MPO | Rbt pAB | DAKO (#A0398) | Citrate pH6 | 1:1000 (1 hr) | TSA568 |
|  | Mx1 | Ms mAB (CL143) | EMD Millipore (#MABF938) | Citrate pH6 | 1:750 (1 hr) | TSA AF647 |
|  | pSTAT1 (Tyr701) | Rbt mAB (58D6) | Cell signaling (#9167S) | Tris pH9 | 1:200 (o/n) | TSA CF750 (1:1500) |
|  | CD163 | Ms mAB (10D6) | Invitrogen (#MA5-11458) | Citrate pH6 | 1:400 (o/n) | TSA AF350 (1:50) |
|  | CD68 | Ms mAB (KP1) | Biocare Medical (#CM033) | Citrate pH6 | 1:400 (o/n) | TSA AF350 (1:50) |
| Anti-inflammatory- CNS | pSMAD3 (phospho S423 + S425) | Rbt mAB (EP823Y) | Abcam (#ab52903) | Tris pH9 | 1:500 (1 hr) | TSA CF750 (1:1500) |
|  | TGF-β1 | Rbt mAB (EPR21143) | Abcam (#ab215715) | DIVA | 1:500 (o/n) | TSA AF568 |
|  | IL-10 | Rbt pAB | Abcam (#ab34843) | Citraconic anhydride | 1:300 (o/n) | TSA AF488 |
|  | Iba1 | Rbt pAB | Biocare Medical (#CP290A) | Citrate pH6 | 1:500 (o/n) | TSA AF647 |
| ROS- CNS | IDO1 | Rbt pAB | Sigma-Aldrich (#HPA023072) | Tris pH9 | 1:1000 (o/n) | TSA CF750 (1:1500) |
|  | SOD1 | Rbt pAB | Sigma-Aldrich (#HPA001401) | Citrate pH6 | 1:1000 (1 hr) | TSA AF647 |
|  | HIF1α | Ms mAB (Halpha111a) | Enzo (#ADIOSA602) | Tris pH9 | 1:100 (o/n) | TSA AF568 |
|  | GFAP | Rbt pAB | Sigma-Aldrich (#HPA056030) | Tris pH9 | 1:1000 (o/n) | TSA AF488 |
| BBB- CNS | IgG | Ms pAB | NHP (#IB3) | Citraconic anhydride | 1:300 (o/n) | TSA AF647 |
|  | CD31 | Shp pAB | R&D Systems (#AF806) | Citrate pH6 | 1:50 (o/n) | AF488 (1:500) |
| CD: cluster of differentiation, DAPI: 4′,6-diamidino-2-phenylindole, GFAP: glial fibrillary acidic protein, HIF1α: hypoxia-inducible factor 1 alpha, Iba1: ionized calcium binding adaptor molecule 1, IDO1: indoleamine 2,3-dioxygenase 1, IgG: immunoglobulin G, IL-10: interleukin 10, Mx1: MX dynamin like GTPase, pSMAD3: phospho-SMAD3, pSTAT1: phospo-STAT1, ROS: reactive oxygen species, SOD1: superoxide dismutase 1, TGFβ1: transforming growth factor beta 1 | | | | | | |
|  |  |  |  |  |  |  |
