# Supplementary material for: Chronic immune activation and gut barrier dysfunction is associated with neuroinflammation in ART-suppressed SIV+ rhesus macaques
Source: PLoS Pathog. 2023 Mar 29;19(3):e1011290. doi: 10.1371/journal.ppat.1011290 (PMC10085024; doi:10.1371/journal.ppat.1011290)
Supplement: S3 Table — (DOCX) [file ppat.1011290.s003.docx]

| **S3 Table. Association of SIV-infected cells in frontal lobe and neuroinflammation** | | | | |
| --- | --- | --- | --- | --- |
|  | **Chronic SIV^b^** | | **VS SIV^c^** | |
| **Parameter** | **P value^a^** | **rho** | **P value^a^** | **rho** |
| vDNA^+^ cells /10^5^ cells |  |  |  |  |
| *Mx-1 area* | 0.973 | -0.014 | 0.279 | -0.382 |
| *pSTAT1 area* | 0.632 | 0.155 | 0.657 | -0.164 |
| *Iba1^+^Mx1^+^ cells* | 0.714 | -0.120 | 0.218 | 0.430 |
| *GFAP^+^Mx1^+^ cells* | 0.304 | 0.324 | 0.179 | 0.467 |
| *pSTAT1^+^ astrocytes* | 0.942 | -0.025 | 0.918 | -0.042 |
| *SOD1^+^ cells* | 0.252 | -0.359 | 0.327 | -0.327 |
| *GFAP^+^SOD1^+^ cells* | 0.294 | -0.349 | 0.330 | -0.346 |
| *Parenchymal IgG* | 0.851 | 0.063 | 0.261 | 0.373 |
| vRNA+ cells /10^5^ cells |  |  |  |  |
| *Mx-1 area* | 0.706 | -0.121 | 0.353 | -0.328 |
| *pSTAT1 area* | 0.088 | -0.516 | **0.033** | -0.690 |
| *Iba1^+^Mx1^+^ cells* | 0.792 | 0.085 | 0.179 | 0.464 |
| *GFAP^+^Mx1^+^ cells* | 0.673 | 0.135 | 0.158 | 0.485 |
| *pSTAT1^+^ astrocytes* | 0.452 | -0.237 | 0.116 | -0.533 |
| *SOD1^+^ cells* | 0.239 | 0.367 | 0.624 | -0.169 |
| *GFAP^+^SOD1^+^ cells* | 0.251 | -0.377 | 0.735 | -0.123 |
| *Parenchymal IgG* | 0.897 | -0.043 | 0.299 | 0.348 |
| BBB: Blood brain barrier; pSTAT1: phosphorylated signal transducer and activator of transcription 1; vDNA: SIV viral DNA; vRNA: SIV viral RNA; VS: virally suppressed SIV+; WM: white matter  ^a^P value and rho determined by non-parametric Spearman correlation (P<0.05 statistically significant)  ^b^ Chronically infected animals were sacrificed following median 53.5 weeks post-infection, median viral load: 5.3 log_10_ SIV RNA copies/mL  ^c^ Virally suppressed animals were treated with ART and sacrificed following median 41 weeks post-infection, median viral load: 1.78 log_10_ SIV RNA copies/mL  Bold values indicate significance (P<0.05), underlined values indicate trend (P<0.1) | | | | |
