# Supplementary figures and images for: Chronic immune activation and gut barrier dysfunction is associated with neuroinflammation in ART-suppressed SIV+ rhesus macaques
Source: PLoS Pathog. 2023 Mar 29;19(3):e1011290. doi: 10.1371/journal.ppat.1011290 (PMC10085024; doi:10.1371/journal.ppat.1011290)

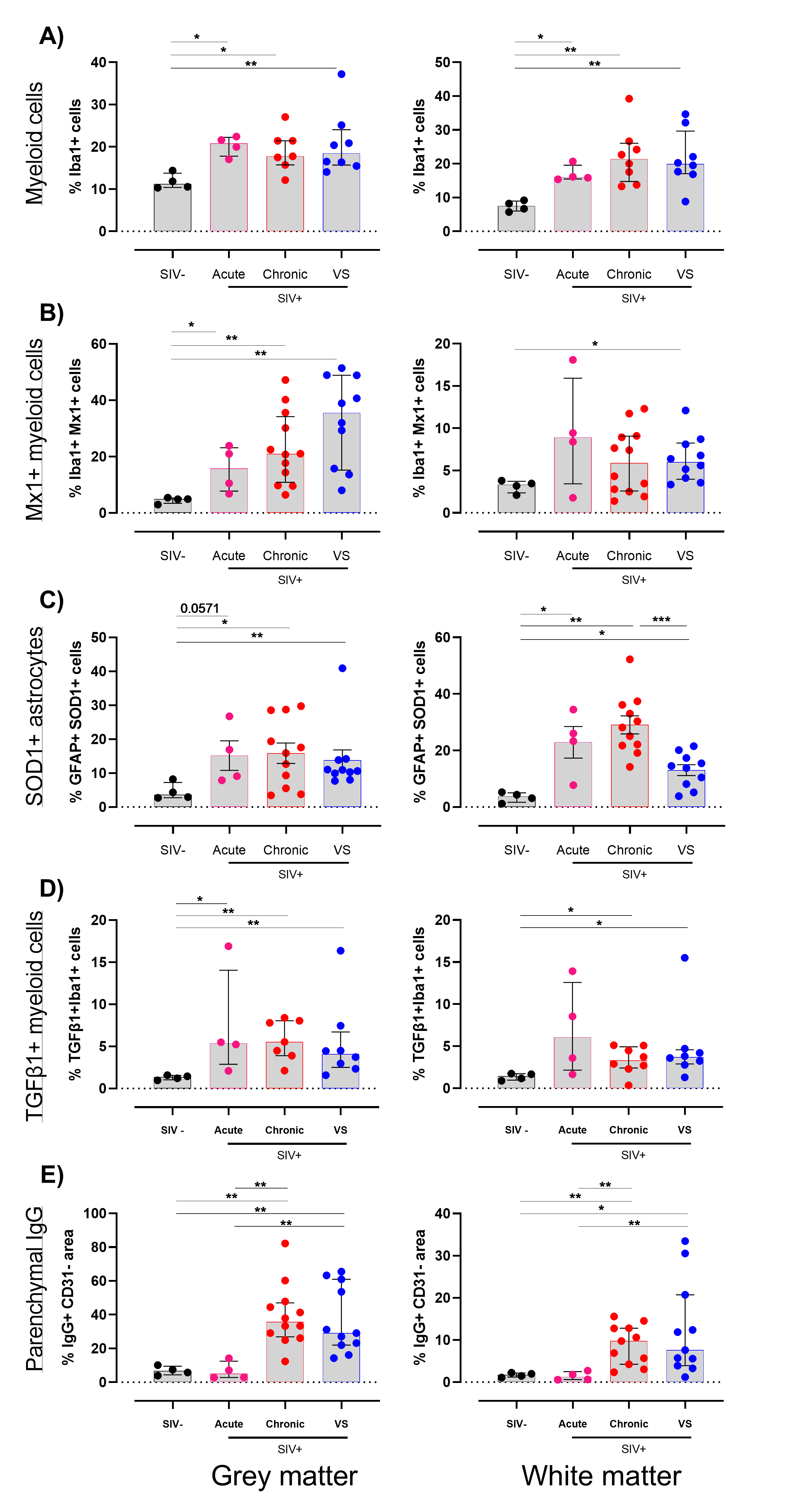

Supplement: S1 Fig — Quantification of (A) myeloid cells (Iba1+), (B) Mx1+ myeloid cells, (C) SOD1+ astrocytes, (D) TGF-β1+ myeloid cells or (E) parenchymal IgG in grey (left panel) or white (right panel) of acute (pink), chronic (red) or virally suppressed (VS) SIV infection or uninfected controls (SIV-). Comparisons made using Mann-Whitney U tests. Median and interquartile ranges shown. *P<0.05; **P<0.01, ***P<0.001. (TIF) [file ppat.1011290.s004.tif]

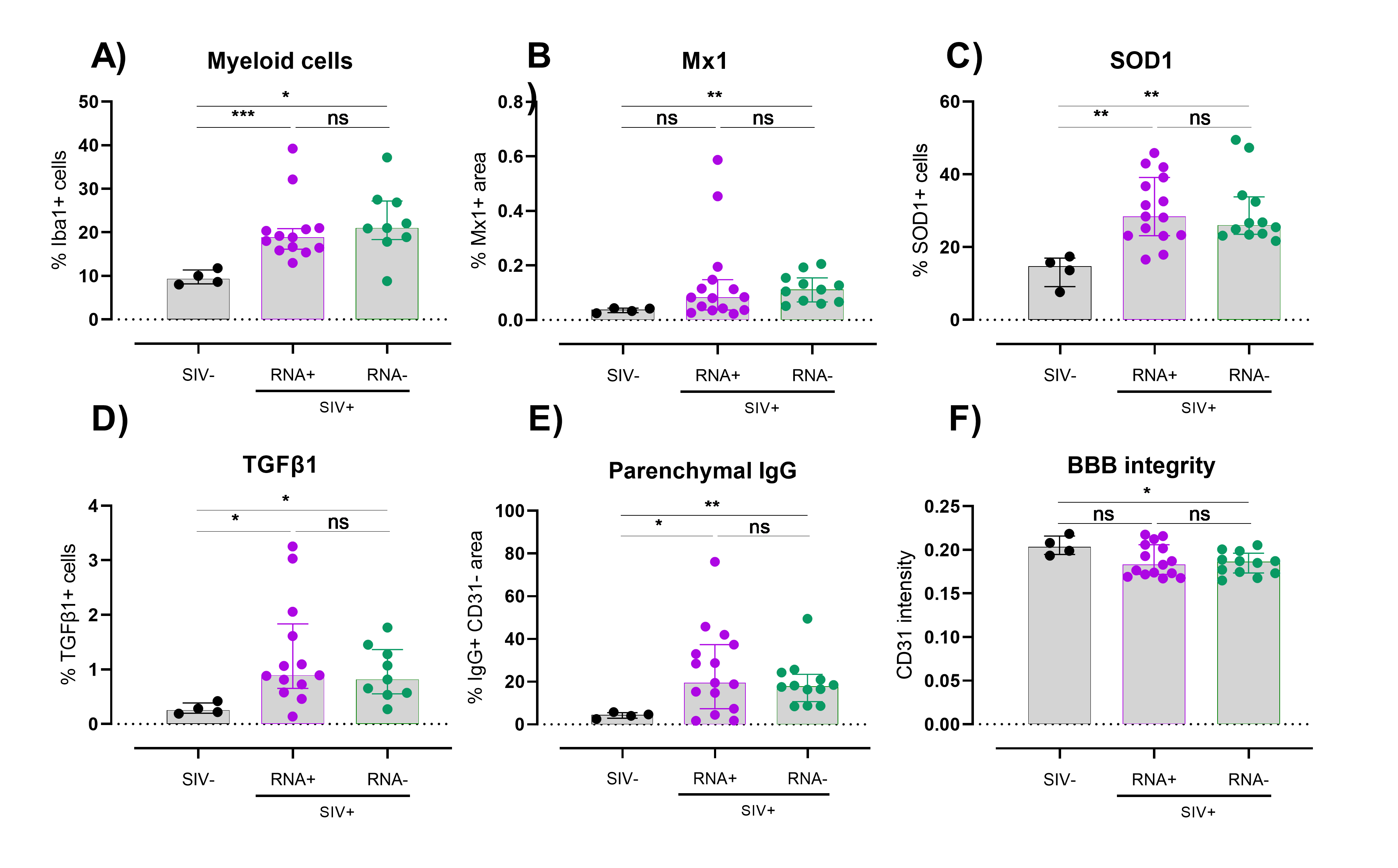

Supplement: S2 Fig — Quantification of (A) Iba1+ myeloid cells, (B) Mx1+ area, (C) % SOD1+ cells, (D) %TGF-β1+ cells, (E) IgG+CD31- area and (F) blood brain barrier (BBB) integrity in the frontal cortex of viremic or virally suppressed SIV+ animals expressing RNA+ (purple) or RNA- (green) in the brain. Comparisons made using Mann-Whitney U tests. Median and interquartile ranges shown. *P<0.05; **P<0.01, ***P<0.001. (TIF) [file ppat.1011290.s005.tif]

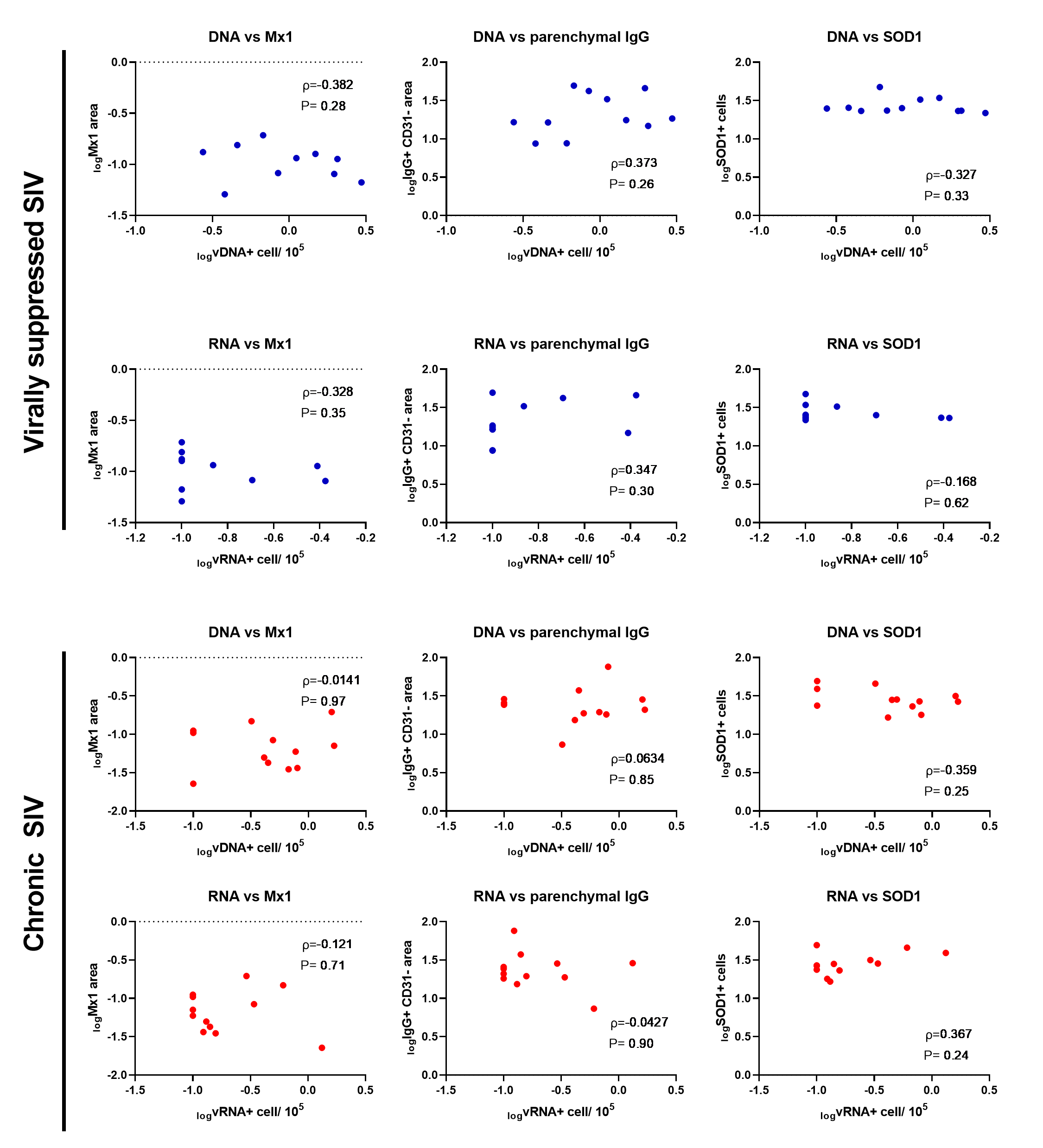

Supplement: S3 Fig — Correlative analysis of SIV vRNA or DNA+ cells and levels of immune activation (Mx1+ area or SOD1+ cells) or blood brain barrier dysfunction (parenchymal IgG+ area) quantified by multiplex immunofluorescence and DNA/RNAscope in frontal cortex tissue of virally suppressed (upper panels; blue; n = 10–11) or chronically SIV-infected rhesus macaques (lower panels; red; n = 11–12). All data log transformed. Samples with undetectable SIV RNA or DNA denoted -1 log vRNA+ (or DNA, as appropriate) cells/105. Non-parametric Spearman’s rho (ρ) and P values shown. P<0.05 considered statistically significant. (TIF) [file ppat.1011290.s006.tif]

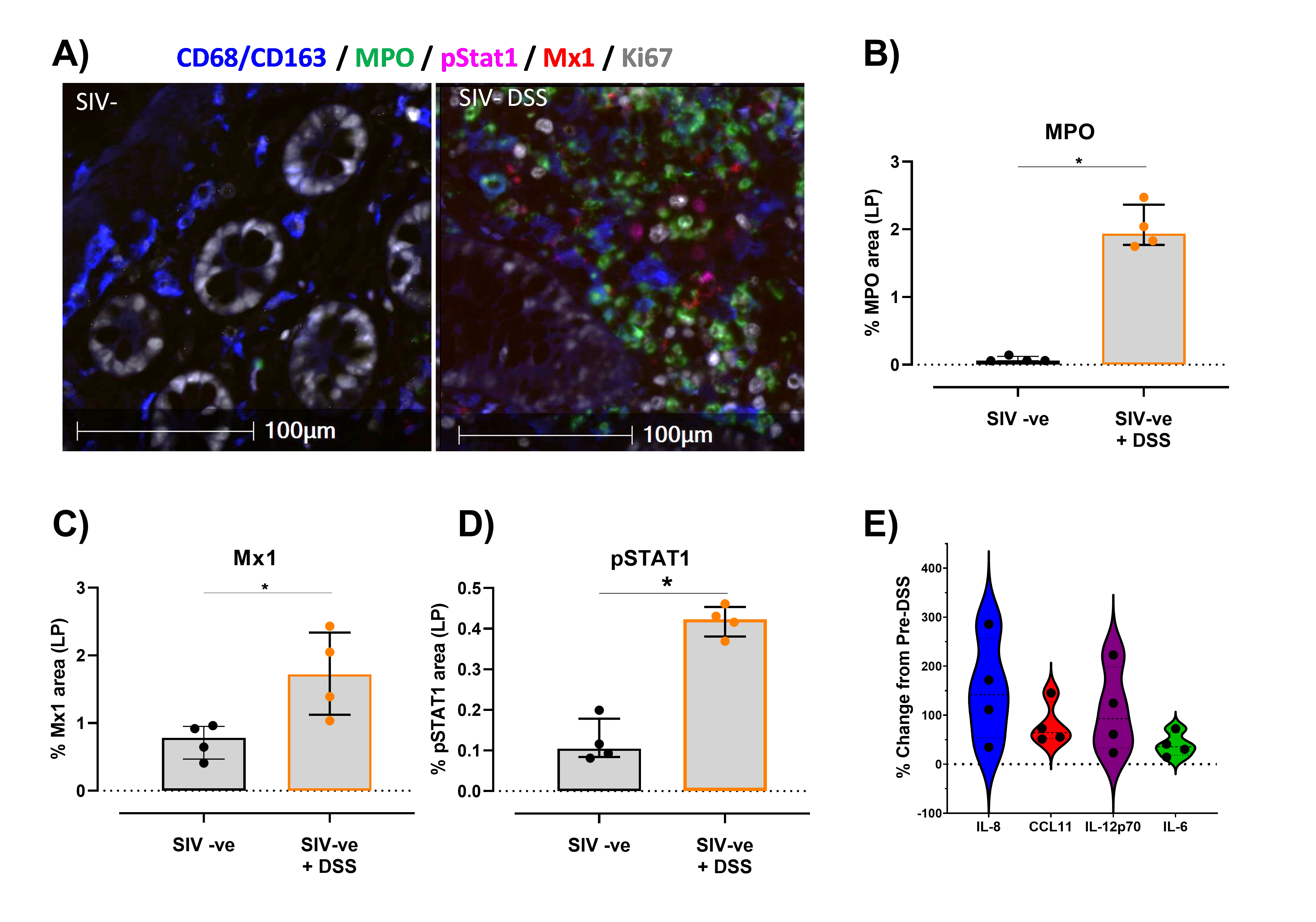

Supplement: S4 Fig — (A) Representative multiplex immunofluorescence images of gut immune activation from SIV-uninfected rhesus macaques either treated with dextran sodium sulfate (DSS; right panel) or left untreated (left panel). Tissue labelled with CD68/CD163 (blue), myeloperoxidase (MPO; green), pSTAT1 (magenta), Mx1 (red) or Ki67 (grey). Scale bars 100 μm. Quantification of the frequency of (B) MPO, (C) Mx1 or (D) pSTAT1 expressing cells between DSS treated (n = 4) or untreated (n = 4) animals. Comparisons made using non-parametric Mann-Whitney U tests, median and interquartile ranges shown. (E) Longitudinal quantification of IL-8, CCL11, IL-12p70 and IL-6 in the plasma of SIV-uninfected rhesus macaques pre- and post-DSS treatment (n = 4) by Luminex immunoassay. Percent change from untreated controls shown. * P<0.05. (TIF) [file ppat.1011290.s007.tif]
